# The Landscape of CAR-T Cell Clinical Trials against Solid Tumors—A Comprehensive Overview

**DOI:** 10.3390/cancers12092567

**Published:** 2020-09-09

**Authors:** Niels Schaft

**Affiliations:** 1 Department of Dermatology, Universtitätsklinikum Erlangen, Friedrich-Alexander-Universität, Erlangen-Nürnberg, Hartmannstraße 14, 91052 Erlangen, Germany; niels.schaft@uk-erlangen.de; Tel.: +49-9131-853-1127; 2 Comprehensive Cancer Center Erlangen European Metropolitan Area of Nuremberg (CCC ER-EMN), Östliche Stadtmauerstraße 30, 91054 Erlangen, Germany; 3 Deutsches Zentrum Immuntherapie (DZI), Ulmenweg 18, 91054 Erlangen, Germany

**Keywords:** CAR-T cell, solid tumor, clinical trial, clinicaltrials.gov, CAR format, tumor antigen, CAR transfer, suicide switch

## Abstract

**Simple Summary:**

Certain immune cells, namely T cells, of cancer patients can be genetically manipulated to express so-called chimeric antigen receptors (CARs), which enables these cells to kill the tumor cells after recognition by the receptor. This therapy is very successful in the treatment of hematologic tumors such as lymphoma or leukemia. However, tumors growing as a solid mass are less susceptible to this kind of treatment. This review summarizes known data of all clinical trials using this therapy against solid tumors that are registered at clinicaltrials.gov.

**Abstract:**

CAR-T cells showed great potential in the treatment of patients with hematologic tumors. However, the clinical efficacy of CAR-T cells against solid tumors lags behind. To obtain a comprehensive overview of the landscape of CAR-T cell clinical trials against this type of cancer, this review summarizes all the 196 studies registered at clinicaltrials.gov. Special focus is on: (1) geographical distribution; (2) targeted organs, tumor entities, and antigens; (3) CAR transfer methods, CAR formats, and extra features introduced into the T cells; and (4) patient pretreatments, injection sites, and safety measurements. Finally, the few data on clinical outcome are reported. The last assessment of clinicaltrials.gov for the data summarized in this paper was on 4 August 2020.

## 1. Introduction

T cells reprogrammed with a tumor specificity via the expression of a chimeric antigen receptor (CAR-T cells) are increasingly used in the adoptive cellular therapy of cancer. The advantage of transferring a CAR, in contrast to normal T-cell receptors (TCRs), is that a CAR can recognize the tumor in an MHC-independent way. The CAR concept was originally developed by Zelig Eshhar (Weizmann Institute of Science, Rehovot, Israel) in the late 1980s [1,2]. Most CARs are created by assembling a tumor-antigen-binding, antibody-derived single chain Fv (scFv) and the intracellular part of the CD3ζ chain linked in cis with one or several co-stimulatory domains [3], however many other formats exist (see below). This modular composition allows for T-cell activation in response to antigens located on the surface of malignant cells by binding of the scFv and subsequent signaling through the CD3ζ chain and co-stimulatory domains [3].

CD19-specific CAR-T cells induced impressive clinical regressions of leukemias or lymphomas in several clinical trials [4,5,6]. This resulted in the approval by the FDA and EMA of Kymriah^®^ (Tisagenlecleucel, Basel, Switzerland), for the treatment of B-cell acute lymphoblastic leukemia (ALL), and Yescarta^®^ (Axicabtagen-Ciloleucel, Santa Monica, CA, USA), for the treatment of aggressive B-cell non-Hodgkin lymphoma [3].

Most clinical trials focus on the elimination of such hematologic tumors; the development of CAR-T cells against solid tumors lags behind (reviewed in [7,8,9,10]). This is due in part to the lack of suitable tumor-specific antigens that can be targeted by CAR-T cells, causing potential on-target/off-tumor toxicity due to the accidental killing of non-malignant bystander cells co-expressing the target antigen [11,12].

This review gives a comprehensive overview of all clinical trials applying CAR-T cells against solid tumors registered at clinicaltrials.gov, irrespective of their status (i.e., withdrawn; suspended; terminated; completed; active, but not recruiting; recruiting; not yet recruiting). The search terms used at clinicaltrials.gov were: “CAR”, “T-body”, “designer T cells”, and “NKR-2”. Just the search term “CAR” already resulted in 1500 found studies, from which CAR-T cell trials against hematologic tumors, long-term follow-up, and retrospective studies, and trials not related to chimeric antigen receptors (e.g., automotive-related studies) were filtered out. To date (last assessment: 4 August 2020), there are 196 CAR-T cell trials against solid tumors registered.

**Figure 1:**
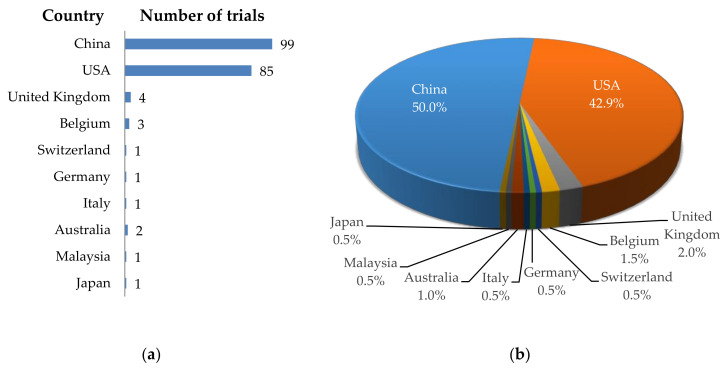
Schematic overview of the geographical distribution of clinical trials using CAR-T cells against solid tumors. (**a**) Number of clinical trials per country; (**b**) Proportional distribution of clinical trials per country. Data was extracted from clinicaltrials.gov.

Looking at the geographical distribution of the registered clinical trials, it is clear that most of these trials are performed in China (*n* = 99; 50.0%; Figure 1), followed by the USA (*n* = 85; 42.9%; Figure 1), and only very few trials are taking place in Europe, Australia, and the rest of Asia (all together responsible for *n* = 14; 7.1%; Figure 1).

Data considering (1) targeted antigen, (2) targeted tumor, (3) CAR format, (4) transfer method of the CAR into the T cells, (5) additionally introduced qualities of the CAR-T cells, (6) number of cells applied, (7) patient pretreatment, (8) clinical outcome, (9) adverse events, and several other parameters are summarized in the following chapters. Additional information on e.g., clinical outcome of the trials and adverse events was gathered through literature search on pubmed.ncbi.nlm.nih.gov [13,14,15,16,17,18,19,20,21,22,23,24,25,26,27,28,29,30].

## 2. CAR-T Cell Clinical Trials against Solid Tumors—Organs, Tumor Entities, Antigens

### 2.1. Targeted Organs

**Figure 2:**
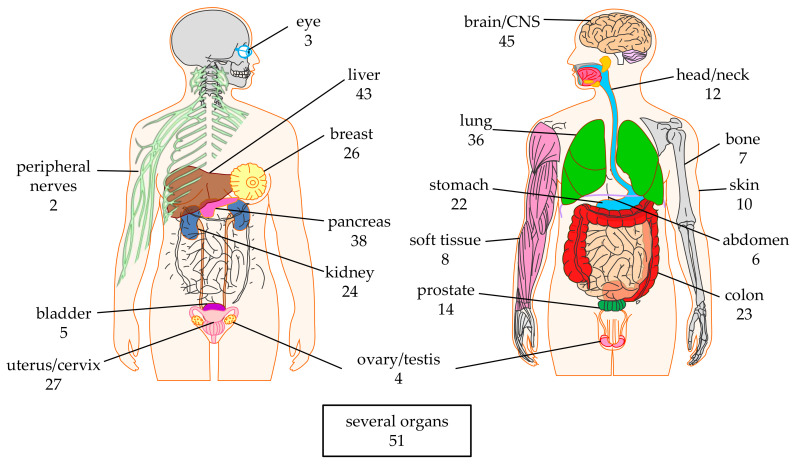
Schematic overview of the organs targeted by CAR-T cells against solid tumors. The numbers indicate the number of clinical trials targeting this organ. Data was extracted from clinicaltrials.gov. The Motifolio Scientific Illustration Toolkit was used for the generation of this figure.

Many different solid tumors (see Section 2.2) are targeted in a total of 20 organs (Figure 2). Especially the tumors in the brain/CNS, liver, pancreas, and lung are targeted in many clinical trials (*n* = 45, 43, 38, and 36, respectively). This might represent the high medical need and/or the absence of effective alternative therapies (i.e., not CAR-T therapies) for tumors in these organs. In total 51 clinical trials target several organs (Figure 2), mostly because the antigen targeted by the CAR-T cells (see Section 2.3) is expressed on tumors in different organs (e.g., epidermal growth factor receptor (EGFR), natural killer group 2D (NKG2D)-ligands, human epidermal growth factor receptor 2 (HER2), mucin 1 (MUC1), and carcinoembryonic antigen (CEA)).

### 2.2. Targeted Tumor Entities

**Figure 3:**
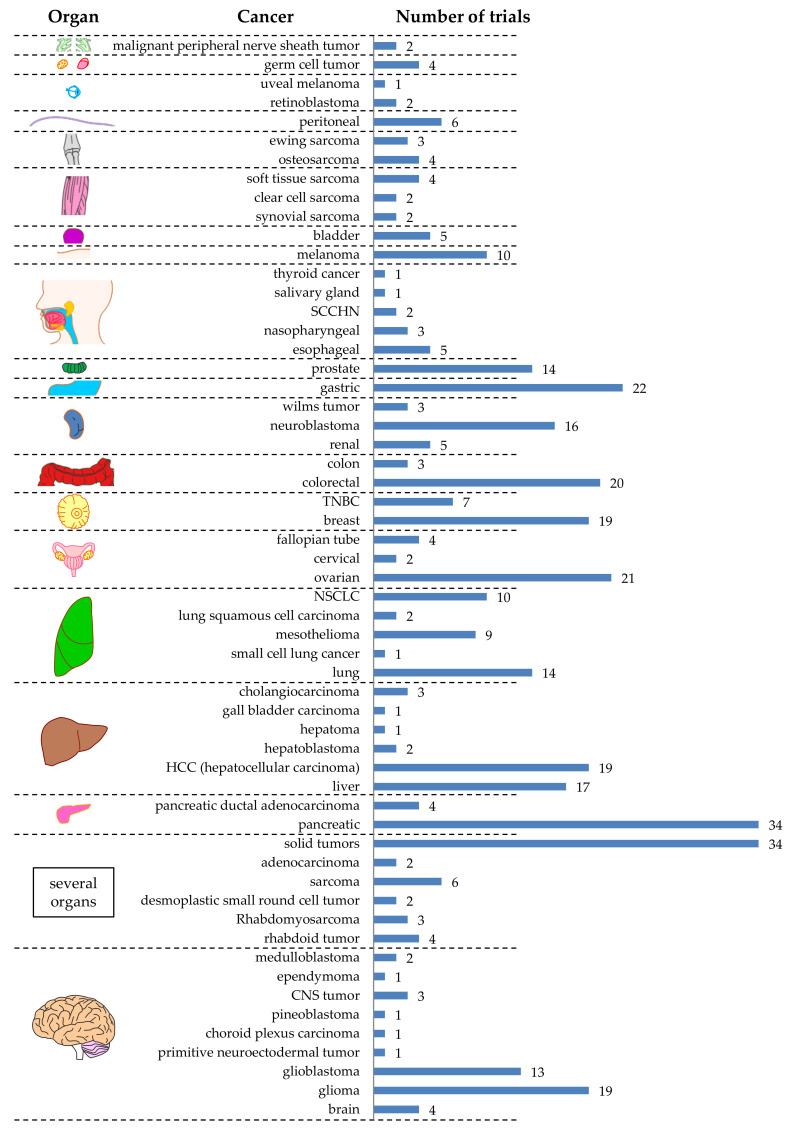
Schematic overview of the tumor entities targeted by CAR-T cells against solid tumors grouped by organ. The numbers indicate the number of clinical trials targeting this tumor. Data was extracted from clinicaltrials.gov. The Motifolio Scientific Illustration Toolkit was used for the generation of parts of this figure.

As can be seen in Figure 3, there are 57 different tumor entities targeted by CAR-T cells registered at clinicaltrials.gov. Nine different tumor entities were described in the brain, six in the liver, and five in the lung (Figure 3). Unfortunately, many registered clinical trials did not exactly specify which tumor entity was targeted. These files just indicated the organ (e.g., “brain”; not specifying which type of tumor) (Figure 3). Furthermore, 34 registered trials just indicated “solid tumor” (Figure 3). The four most targeted tumor entities are pancreatic cancer (*n* = 34), gastric cancer (*n* = 22), ovarian cancer (*n* = 21), and colorectal cancer (*n* = 20) (Figure 3). This does not reflect the world-wide cancer incidence. In 2018, the top 3 of cancer types newly diagnosed for both sexes was: (1) lung cancer (12.3%), (2) breast cancer (12.3%), and (3) colorectal cancer (10.6%) [31,32,33]. This could be caused by local difference in cancer incidence (e.g., in China, gastric cancer is the third most diagnosed cancer after lung cancer and colorectal cancer, and even the second most common cause of cancer-related death [34], and might therefore have a higher interest in performing clinical trials targeting this cancer entity). Indeed, of the 22 clinical trials targeting gastric cancer, 15 were/are performed in China.

At which tumor stage the CAR-T cells are applied, i.e., at an early stage (e.g., only primary tumor present), or at a late stage with several (distant) metastases, can have an impact on the effectiveness of CAR-T-cell therapy. However, most registered trials do not provide information on the exact treated tumor stage. One can hypothesize that the therapy would most probably be more effective at lower tumor burden.

### 2.3. Targeted Antigens

Ideal target antigens on solid tumors unify three essential attributes: (i) uniform presence on the surface of malignant cells reducing the risk for antigen-negative escape variants; (ii) absent expression on non-malignant host cells precluding on-target/off-tumor activity, which harbors the potential for severe, potentially lethal, side-effects [12]; and (iii) crucial role as an oncogenic driver in cancer cells, which may compound antigen-shutdown due to the selective survival advantage conferred on malignant cells. Co-expression on by-stander cells maintaining the tumor-microenvironment—such as tumor-associated vasculature, fibroblasts, and macrophages—represents another beneficial trait.

The registered CAR-T cell studies at clinicaltrials.gov target 44 different antigens on solid tumors (Table 1). Table 2 shows a more detailed overview of which antigen is targeted on which tumor. Sixteen clinical trials target several antigens at the same time [35,36], and two clinical trials do not disclose the targeted antigen (Table 1). The top six targeted antigens that are expressed on solid tumors in many different organs are (1) EGFR [37,38,39,40,41] (14 different organs), (2) NKG2D-ligands [42,43] (11 different organs), (3) HER2 [12,44,45,46,47,48,49,50] (11 different organs), (4) B7-H3 (10 different organs), (5) MUC1 (9 different organs), and (6) CEA [51,52,53,54,55,56,57,58] (9 different organs) (Table 1).

EGFR and HER2 are members of the ErbB family of receptor tyrosine kinases (i.e., EGFR (ErbB-1), HER2/(neu) (ErbB-2), Her 3 (ErbB-3), and Her 4 (ErbB-4). Mutations in EGFR lead to its overexpression, which results in its constant activation and uncontrolled cell division in many different cancers (e.g., non-small cell lung cancer (NSCLC), colorectal cancer, pancreatic cancer, etc.) [59,60,61]. HER2 functions similarly and is overexpressed mainly in breast cancer, but also in other cancer types like ovarian cancer, glioma, and many more [62,63,64].

The NKG2D-ligands MIC-A, MIC-B, and the ULBPs 1, 2, 3, 4, 5, 6 are induced-self proteins, which are upregulated on stressed, infected, and transformed cells. These ligands can be recognized by the NKG2D receptor expressed on NK cells, NKT cells, γ/δ T cells, and activated CD8^+^ αβ T cells [65,66]. Colorectal cancers, ovarian cancers, and other cancers [67,68,69,70] express higher levels of NKG2D-ligands and can be targeted by CAR-T cells incorporating NKG2D in the chimeric receptor.

B7-H3 (i.e., CD276, or B7 homolog 3) is a co-stimulatory molecule for T cells and is for example expressed on activated dendritic cells and monocytes. T cells stimulated by B7-H3 proliferate and differentiate into cytotoxic T cells and selectively secrete IFNγ when TCR signaling and B7-H3 co-stimulation are combined [71]. It has only a limited expression on healthy tissues [72,73]. However, B7-H3 is overexpressed on neuroblastomas, where it inhibits recognition and killing of the tumor cells by NK cells [74]. Furthermore, CD276 is overexpressed on several other tumors—such as pancreatic ductal adenocarcinoma (PDAC), prostate cancer, ovarian cancer, lung cancer, and clear cell renal carcinoma—and on tumor-associated vasculature and stroma fibroblasts [73,75,76,77,78,79,80,81,82,83,84,85].

Mucin 1 (MUC1) is a highly glycosylated membrane protein expressed on the surface of epithelial cells in intestine, stomach, lung, eye, and other organs, where it inhibits pathogens from reaching the cell membrane by binding them to oligosaccharides [86,87]. MUC1 is overexpressed on colorectal, breast, ovarian, lung, and pancreatic cancers [88,89,90].

Carcinoembryonic antigen (CEA, also CEACAM5) is a glycoprotein which is widely expressed during fetal development and on some adult tissues (e.g., epithelium of the colon, stomach, and esophagus) [91]. In normal epithelial cells of the lung and gastrointestinal tract, CEA has an apical polarity and is facing the lumen and cannot be recognized by CAR-T cells [92]. Its function and signaling in normal tissue are still not fully understood [93]. CEA is overexpressed in colorectal, pancreatic, gastric, lung, and breast carcinoma where it plays a role in metastasis of the tumor [94]. In carcinomas, CEA has lost its apical polarity and is even partly shed, resulting in an increased serum level [95]. At this stage, CEA expressed on tumor cells can be targeted by CAR-T cells [96].

These six antigens are not perfectly tumor specific. When looking and mRNA and/or protein expression on BioGPS (www.biogps.org [97]), The Human Protein Atlas (www.proteinatlas.org [98]), and Expression Atlas (www.ebi.ac.uk/gxa/), all of these antigens are expressed to some extend on some normal tissues. It is very hard to find a target antigen for CARs on solid tumors that is not expressed on healthy tissue, and the use of CAR-T cells is a double-edged sword, because the potency of these cells can also turn against the patient [99]. It can never be excluded that some rare but important cell type in healthy tissue expresses the antigen. A case highlighting the lethal potential associated with on-target/off-tumor toxicity was shared by investigators from the NCI. Shortly after infusing T cells expressing an HER2-specific CAR to a patient with metastatic colon cancer, clinical symptoms of acute respiratory distress syndrome were observed necessitating mechanical ventilation [12]. Unfortunately, the patient died 5 days later [12]. The cause of death was assumed to be on-target/off-tumor toxicity elicited by low levels of HER2 on epithelial cells in the lung. Remarkably, the CAR was derived from the FDA-approved monoclonal antibody trastuzumab, which has been widely used without the occurrence of severe pulmonary toxicities [100]. This justifies the addition of safety measurements when using CAR-T cells in patients (see Section 4.3).

## 3. CAR-T Cell Clinical Trials against Solid Tumors—CAR Transfer Methods, CAR Formats, Extra Features

### 3.1. Transfer Methods to Introduce the CAR into T Cells

**Figure 4:**
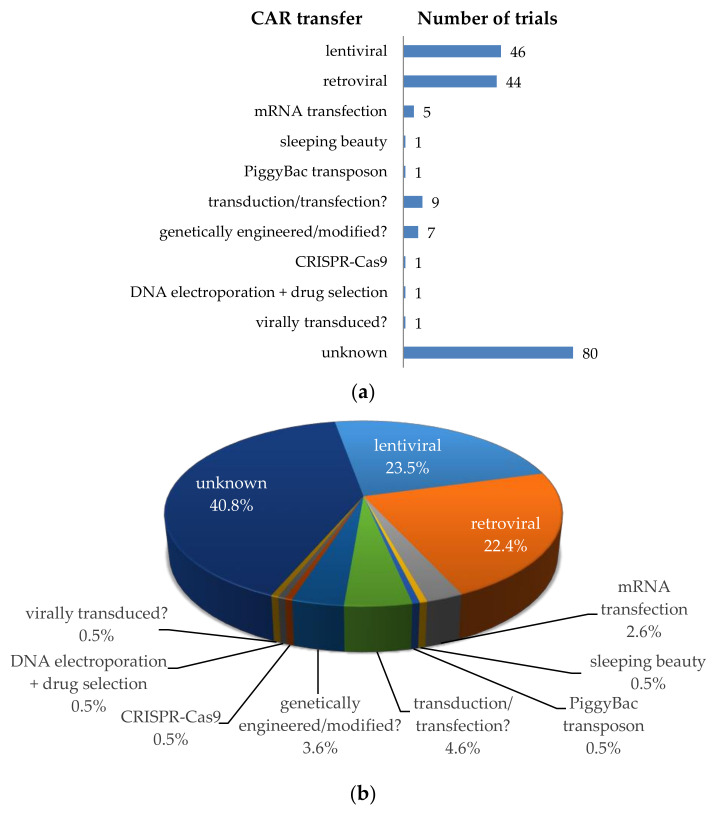
Schematic overview of the methods used for CAR transfer into T cells. (**a**) Number of clinical trials using a specific transfer method; (**b**) Proportional distribution of clinical trials per transfer method. Data was extracted from clinicaltrials.gov.

To introduce the chimeric antigen receptors into T cells, several methods can be used (Figure 4). Most clinical trials use a viral transfer method (retroviral or lentiviral) to stably introduce the CAR. During this procedure, a CAR encoding gene is transported by the virus into the T cell, where it is stably integrated into the genomic DNA. The offspring of these transduced cells will all carry the CAR gene and can express the receptor on its cell surface. Some disadvantages of viral transduction are the random integration into the host cell’s genome, which can result in destruction or activation of some genes (i.e., insertional mutagenesis), and the introduction of viral material/genes. This method can cause problems in CAR-T cell treated patients. Lamers et al. described, for example, the development of immune responses to the receptor-encoding transgene and the retroviral vector [101]. As can be seen in Figure 4, lentiviral [102] and retroviral [103,104] transduction was mostly used for the transfer of the CAR (i.e., *n* = 46; 23.5%, and *n* = 44; 22.4%, respectively). Unfortunately, most clinical trial registrations do not clearly indicate which transfer method is used (i.e., transduction/transfection? *n* = 9, genetically engineered/modified? *n* = 7, virally transduced? *n* = 1, or no indication at all (unknown) *n* = 80; Figure 4). Some clinical trials use a non-viral gene delivery system or a transfer method integrating the CAR gene into a specified site (i.e., sleeping beauty transposon system [103,105,106,107], PiggyBac transposon system [103,107], CRISPR-Cas9 [108], or transfection of DNA or RNA [109]). The latter two methods do not result in an integration of the CAR-encoding gene into the host cell’s genome, which has certain advantages (highlighted in Section 4.3 for mRNA transfection).

The time needed for the production of the CAR-T-cell product is highly variable and can even be patient dependent. Most registered clinical trials do not provide details on the production time. However, it is usually several weeks.

### 3.2. CAR Formats; the Classical and the More Exotic Models

#### 3.2.1. CARs: The Classical Models

**Figure 5:**
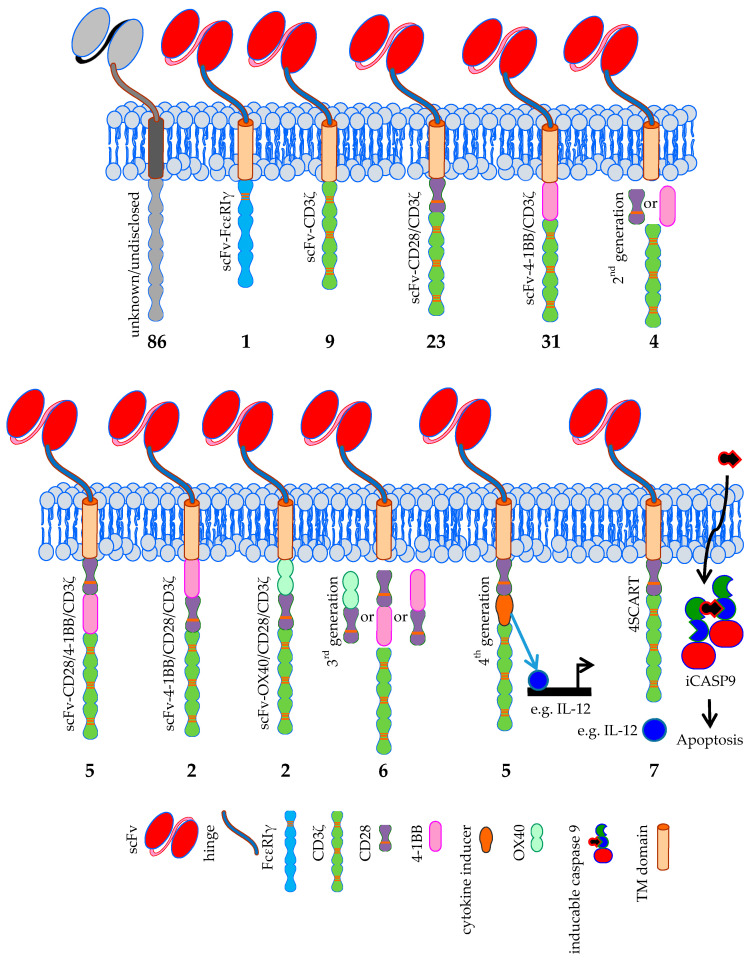
Schematic overview of the classical CAR formats used in clinical trials treating solid tumors. The number of clinical trials using a specific CAR format is indicated. Data was extracted from clinicaltrials.gov. TM = transmembrane domain, iCASP9 = inducible caspase 9, 4SCART = fourth generation safety CAR-T cells. The Motifolio Scientific Illustration Toolkit was used for the generation of parts of this figure.

Since the first CAR concept was presented by Zelig Eshhar in 1989 [1,2], several generations of CARs were developed. The classical CAR always incorporates an antibody-based scFv, which binds to the tumor antigen. In first generation CARs, this scFv is linked via a flexible linker and transmembrane domain to either the intracellular signaling domain of FcεRIγ or CD3ζ [110]. In clinicaltrials.gov there are indeed one trial registered using the first signaling domain and nine trials using CD3ζ (Figure 5). Most registered trials, however, use a second generation CAR [110] incorpora-ting a co-stimulatory domain. Co-stimulation is mostly provided by CD28 or 4-1BB domains [3].

Physiologically, CD28 co-stimulation promotes the production of IL-2, -6, -10, and further interleukins, as well as cell cycle progression, survival, differentiation, and cytolytic functions [111]. Many studies that employed CARs with a CD28 signaling domain observed potent and quick anti-tumor effector functions. However, these were short-lived and associated with limited cell persistence in vivo when compared to, e.g., the 4-1BB signaling domain. Notwithstanding, it was shown that human CD8^+^ CAR-T cells containing a CD28 co-stimulatory domain differentiate towards both a central-memory and effector-memory type [112,113,114,115,116,117,118,119]. The transmembrane domain of CD28 used in many CARs as a connector between extra- and intra-cellular domains is associated with improved expression of these CARs on the surface [120,121], but might also cause tonic CAR signaling [122,123] and thereby lead to Fas-dependent activation-induced cell death (AICD) in CAR-T cells, possibly explaining the observed limited cell persistence [113]. Clinical trials confirmed the preclinical findings that CD28 supports strong but short-lived anti-tumor efficacy [124,125].

Physiological 4-1BB signaling in T cells enhances cell cycle progression and proliferation, cytokine secretion, cytolytic potential, and inhibits clonal deletion and AICD [126,127]. CARs containing 4-1BB as a signaling domain allowed for a more robust cell activation, as well as an increased persistence in vivo, and 4-1BB co-stimulation promotes differentiation of CAR-T cells towards a central-memory phenotype [4,112,113,114,115,116,117,118,128,129]. However, 4-1BB co-stimulated CARs showed a slower onset of cytotoxicity, but longer durability and accumulation of CAR cells over time [128]. Potent anti-tumor efficacy and very long persistence of 4-1BB-containing CAR-T cells in patients were also reported in clinical trials [4,130].

CD28 is incorporated in CARs used in 23 trials [131,132], and 4-1BB co-stimulation is used in 31 CAR-T cell trials (Figure 5). Four trials just indicate that second generation CARs are used, but do not specify which co-stimulatory domain is included (Figure 5). In 86 registered trials, the used CAR format is not disclosed (Figure 5).

The third generation CARs [111] used in the registered clinical trials incorporate combinations of CD28/4-1BB, 4-1BB/CD28, or OX40/CD28 co-stimulatory domains (Figure 5) [133,134]. Six trials mention the use of third generation CARs but do not indicate the exact co-stimulatory domains used, or their order in the chimeric molecules (Figure 5). Fourth generation CARs (also known as “T cells redirected for universal cytokine-mediated killing” (TRUCKs)) are in principle second generation CARs with the extra feature that they can induce the production of e.g., cytokines in a very restricted local fashion [135]. The effects induced depend on the cytokines that are secreted: e.g., IL-12 can activate an innate immune response in the tumor [136], causes less susceptibility to Treg suppression [137], and increases cytokine secretion and expansion [138,139], and IL-15 increases the anti-tumor activity of the CAR-T cells [140]. A variant of this format is the 4SCART (fourth generation safety CAR-T) which additionally incorporates an inducible caspase 9 as a safety measurement (will be described in detail in Section 4.3). Five registered trials rely on the fourth generation CAR format, while seven trials use the 4SCART format (Figure 5).

#### 3.2.2. CARs: The More Exotic Models

**Figure 6:**
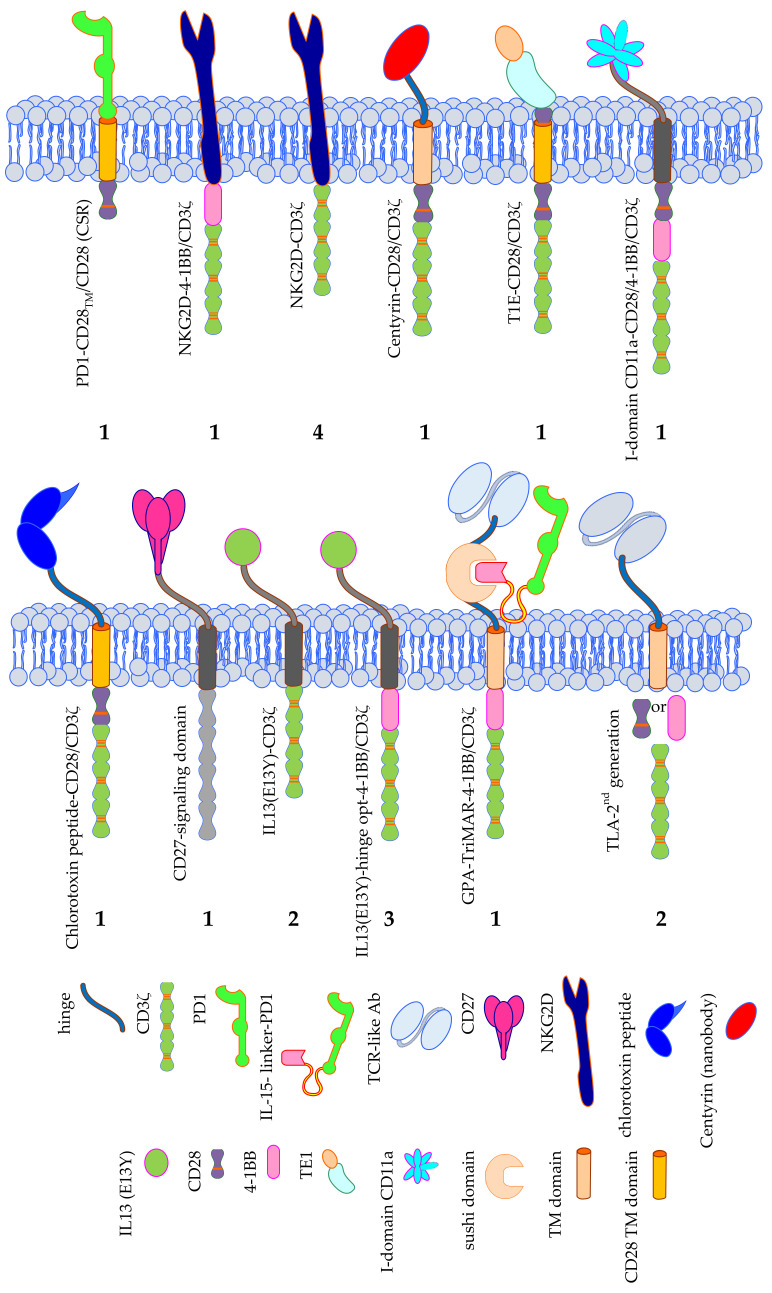
Schematic overview of the exotic CAR formats used in clinical trials treating solid tumors. The number of clinical trials using a specific CAR format is indicated. Data was extracted from clinicaltrials.gov. TM = transmembrane domain, PD1 = programmed cell death protein 1, IL13 (E13Y) = mutated IL-13 optimized to bind IL-13Rα2, TE1 = promiscuous ErbB ligand derived from EGF and TGFα. The Motifolio Scientific Illustration Toolkit was used for the generation of parts of this figure.

A group of 19 clinical trials in total use alternative binding moieties instead of a scFv directed against an antigen expressed on the cell surface of the tumor (Figure 6).

One trial applies a so-called chimeric switch receptor (CSR), consisting of PD1 as extracellular domain and CD28 transmembrane and intracellular signaling domain (Figure 6). As tumors often express PD-L1 on their surface to activate the inhibitory PD1 receptor on T cells to circumvent/inhibit an anti-tumor T-cell response, the CSR will turn the inhibitory into an activation signal induced by CD28 [141].

Several trials target NKG2D ligands by linking NKG2D to either CD3ζ (4 trials), or 4-1BB/CD3ζ (1 trial) (Figure 6) [43]. This strategy is very attractive, since NKG2D binds a plethora of ligands (MIC-A, MIC-B, and the ULBPs 1, 2, 3, 4, 5, 6), that are induced-self proteins, which are upregulated on stressed, infected, and transformed cells, as already described above, and therefore can be used for many types of cancer.

Poseida Therapeutics, Inc., together with the City of Hope Comprehensive Cancer Center, the Sarah Cannon Research Institute at HealthONE, and the Memorial Sloan Kettering Cancer Center, are performing a phase 1/2 trial with CAR-T cells in which the binding moiety consists of a nanobody (named Centyrin) specific for PSMA linked to the signaling domains of CD28 and CD3ζ (Figure 6).

The T1E-CD28/CD3ζ CAR is coupling a promiscuous ErbB ligand derived from EGF and TGFα to a fused CD28/CD3ζ endodomain (Figure 6). This CAR can bind several ErbB2 dimers (i.e., HER2, HER3, and EGFR) and therefore can target several tumors [142,143,144].

AffyImmune Therapeutics, Inc., together with the Weill Medical College of Cornell University is clinically testing AIC100 CAR-T cells. The binding moiety of this CAR consists of the I-domain of CD11a of LFA-1, which binds to ICAM1 on tumor cells. Intracellularly CD28, 4-1BB, and CD3ζ domains facilitate signaling (Figure 6).

Originally used as an imaging agent to guide glioblastoma resection surgery, and to carry different therapeutics to these tumors, a 36-amino acid long peptide of chlorotoxin, a component of scorpion venom, was linked to the transmembrane and intracellular domains of CD28 and signaling domain of CD3ζ to form a CAR (Figure 6). Preclinical testing showed that chlorotoxin binds to a greater proportion of patient tumors, and cells within these tumors, while ignoring non-tumor cells in the brain and other organs, and that this binding to its ligand is not so much influenced by tumor heterogeneity compared to other antigens such as IL13Rα2, HER2, and EGFR [145]. Therefore, the City of Hope Medical Center, together with the National Cancer Institute is now testing this CAR in a clinical trial against recurrent glioblastoma and recurrent malignant glioma.

CD70-CD27 interactions are important for the regulation of adaptive immunity. CD70 shows a restricted expression on non-malignant cells, but is expressed on some solid tumors (e.g., on renal carcinoma, pancreatic cancer, breast cancer, melanoma, and ovarian cancer) and is implicated in tumor escape from immunosurveillance [146,147,148,149,150]. In a clinical trial performed by the NCI, a CAR consisting of CD27, linked to undisclosed intracellular signaling domains is used (Figure 6).

Five clinical trials use a mutated form of IL-13, which is optimized for binding to IL-13Rα2, either linked to the signaling domain of CD3ζ alone, or a combination of 4-BB and CD3ζ signaling domains for the treatment of patients with glioblastoma, glioma, or melanoma (Figure 6) [151,152,153,154].

A highly complex CAR for the treatment of melanoma patients was developed by Timmune Biotech Inc., and used in a clinical trial by the Second Affiliated Hospital of Hainan Medical University. This so-called GPA-TriMAR binds to a peptide derived from the melanoma-associated antigen gp100, presented in HLA-A2 through a TCR-like antibody [155,156] (Figure 6). This might increase the tumor specificity, but nullifies the advantage of a CAR that it can bind to a native cell-surface tumor antigen, which does not need to be processed and presented in an HLA-context and is therefore not dependent on the HLA-type of the patient. The other two extracellular subunits are a sushi domain, which can bind IL-15, and an IL-15-linker-PD1 construct (Figure 6). The latter two subunits are supposed to stimulate the innate immune system. The GPA-TriMAR is linked to the intracellular signaling domains of 4-1BB and CD3ζ [157].

Finally, two clinical trials use a TCR-like antibody (TLA) as binding moiety linked to a second generation intracellular domain (i.e., either CD28/CD3ζ, or 4-1BB/CD3ζ) (Figure 6) [158]. Both trials target a peptide of alphafetoprotein (AFP) presented in HLA-A2 in HCC patients. The two different CARs were both developed by the companies Eureka Therapeutics Inc. and Aeon Therapeutics (Shanghai, China) Co., Ltd. providing only limited published data [158].

### 3.3. Add-Ons; T-Cell Populations Used for Transfer or Extra Features Introduced into CAR-T Cells

#### 3.3.1. T-Cell Populations Used for CAR-T-Cell Therapy

In many clinical trials, certain T-cell populations are used for the CAR introduction. For example, T cells specific for VZV, EBV, adenovirus, CMV, or multivirus-specific T cells are used. The idea behind it is that these T cells can be stimulated via their endogenous virus-specific TCR to proliferate and therefore increase their persistence and number. Epitopes of latent viruses like EBV or CMV are constantly presented and stimulate the CAR-T cells. Another strategy is to use virus vaccination to boost T-cell proliferation (like VZV vaccination, or oncolytic adenovirus injected intratumorally). Some trials used directly vaccine-specific T cells to induce this proliferation [46,159,160,161]. To increase persistence of the CAR-T cells, one can alternatively use memory T cells for the transfer of the CAR [162,163].

Most trials use autologous patient T cells to introduce the CARs. However, there might be certain situations making the use of allogeneic T cells necessary (e.g., not enough T cells can be isolated/expanded from the patient, or CAR-T cell therapy is performed after allogeneic stem cells transplantation). Furthermore, the use of allogeneic T cells that are genetically modified (see below) in such a way that they are not recognized by the endogenous immune system, or can harm healthy tissue of the patient, is very attractive, and can generate an off-the-shelf therapy for many different patients. Alternatively, the endogenous TCR of γ/δ-T cells does not recognize peptides presented in HLA molecules [164,165] and therefore they do not induce graft-versus-host disease after CAR-T-cell transfer in HLA-mismatched patients [166]. This allows the use of γ/δ-T cells for the generation of CAR-T cells from healthy donors, which are not impaired by tumor- or therapy-related immunosuppression [167,168], and the application of CAR-T cells in a multitude of patients, irrespective of their T-cell numbers and HLA-type. An additional positive effect is that γ/δ-T cells have an intrinsic anti-tumor activity [169], potentiating the adoptive T-cell therapy against tumors. Moreover, the number of CAR-γ/δ-T cells can be boosted in vivo by systemic administration of zoledronic acid [170].

#### 3.3.2. Extra Features Introduced into CAR-T Cells

Several resistance mechanisms to CAR-T-cell therapy in solid tumors may play a role in the observed lower effectiveness compared to CAR-T cells in hematologic malignancies [171,172,173,174]. For example, the tumor microenvironment can be hostile for CAR-T cells (e.g., unfavorable pH or oxygen levels) or unfavorable electrolyte or cytokine concentrations, inhibiting an effective immune response [175,176,177]. Additionally, the homing of CAR-T cells can be hampered in solid tumors [172]. Furthermore, solid tumors can induce inhibitory receptors on CAR-T cells like PD1 and CTLA-4, making the CAR-T cells exhausted. The patient’s own immune cells can even attack the CAR-T cells e.g., by antibody production [178,179]. To overcome these resistance mechanisms, several strategies were developed, like induced expression of cytokines, expression of constitutively active or dominant negative cytokine receptors, expression of homing receptors, prevention of anti-CAR antibody production, or blocking of PD1/CTLA-4. All are described in more detail below.

**Figure 7:**
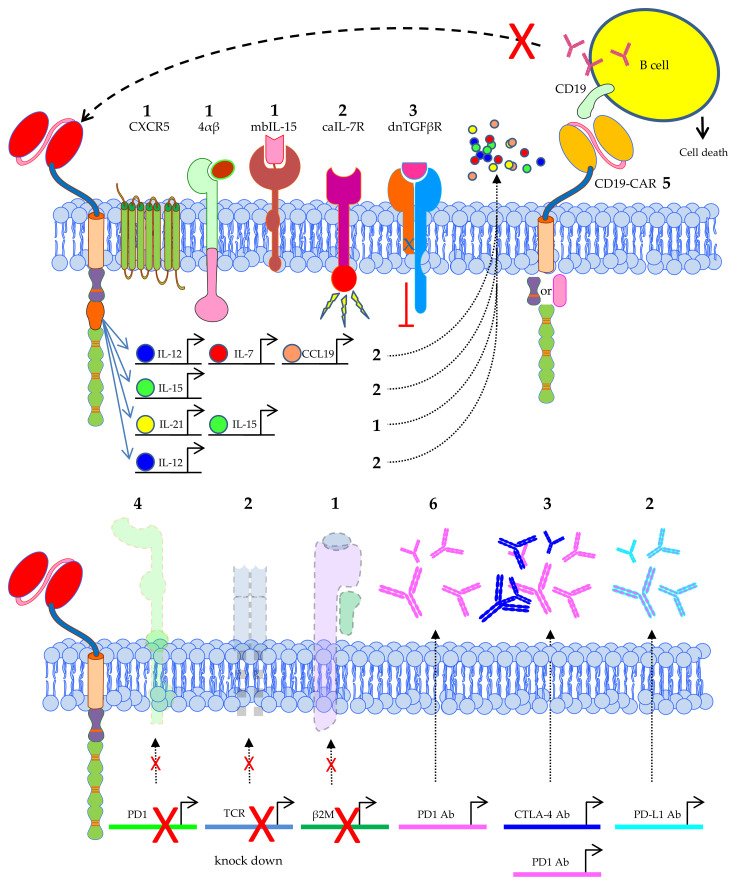
Schematic overview of the extra features introduced into CAR-T cells used in clinical trials treating solid tumors. The number of clinical trials using a specific feature is indicated. Data was extracted from clinicaltrials.gov. 4αβ = chimeric cytokine receptor containing the IL-4Rα ectodomain coupled to the IL-2Rβ endodomain, mbIL-15 = membrane-bound IL-15, caIL-7R = constitutively active IL-7 receptor, dnTGFβR = dominant negative TGFβ receptor. The Motifolio Scientific Illustration Toolkit was used for the generation of parts of this figure.

An often-used strategy to improve the effectiveness of CAR-T cells is to equip them with the ability to secrete cytokines like IL-12, IL-15, IL-21, IL-7, or combinations thereof (Figure 7). In two trials performed by the Second Affiliated Hospital of Guangzhou Medical University and the Sixth Affiliated Hospital of Wenzhou Medical University in China, the used CAR-T cells produce IL-7 and CCL19 [180,181,182,183]. IL-7 is known for its positive effects on T-cell survival [184], and CCL19 is a chemokine attracting other endogenous immune cells, like dendritic cells, B cells, and central memory T cells [185,186,187] to the tumor site. IL-12, IL-15, and IL-21 are all cytokines known to stimulate immune cells. The fact that these cytokines are produced very locally is an advantage, since some of them can have toxic effects when applied systemically [135].

Some trials take this even a step further and use CAR-T cells co-expressing a constitutively active IL-7 receptor [188], or a membrane-bound form of IL-15 [189] (Figure 7). Others introduce a chimeric cytokine receptor containing the IL-4Rα ectodomain coupled to the IL-2Rβ endodomain (4αβ) resulting in a robust expansion of CAR-T cells after IL-4 binding, a cytokine with several pathophysiologic and therapeutic links to cancer [190] (Figure 7). TGFβ is known to have an immunosuppressive effect, and many tumors, especially prostate cancer, secrete TGFβ and thereby promote metastasis and neoangiogenesis and suppress T cells [191,192]. In vitro and in vivo models showed that blocking TGFβ signaling in T cells by using a dominant-negative TGFβ receptor II (i.e., a truncated form which lacks the intracellular domain necessary for downstream signaling) [193] resulted in an increased ability to infiltrate, proliferate, and mediate anti-tumor responses [194]. Therefore, this dnTGFβR was co-introduced next to a CAR specific for PSMA in T cells to treat prostate cancer patients [195] (Figure 7).

CAR-T cells can also be engineered to express homing molecules to target these cells to specific tissue locations. A clinical trial performed by the Sun Yat-sen University, Guangzhou, China in collaboration with Bio-gene Technology Co., Ltd., Guangzhou, China uses CAR-T cells specific for EGFR which were additionally transduced with the lymphoid follicle homing molecule CXCR5 [40]. Alternatively, to circumvent problems with homing of CAR-T cells to the tumor site, these cells can be directly infused into the tumor [55,154,196,197].

In total, five clinical trials co-introduce an anti-CD19 CAR next to the tumor-antigen-specific CAR into T cells. This extra CAR is directed against CD19 positive B cells which can produce antibodies, and these cells will be lysed. That means that CD19-positive cells that might produce anti-CAR antibodies, e.g., because the tumor-antigen-specific CAR is based on a murine scFv, will also be destroyed. This will increase the persistence of the anti-tumor CAR-T cells in the patients (Figure 7). Additionally, lymphodepleting chemotherapy will also prevent graft rejection.

The anti-tumor activity of T cells can be inhibited by various tumor-associated immunosuppressive ligands like PD1 and CTLA-4 [198]. Several strategies are used in clinical trials to prevent inhibition of the CAR-T cells by PD1 or CTLA-4 interactions between tumor cells and CAR-T cells. Four clinical trials knockout the PD1 gene in the CAR-T cells (Figure 7), preventing the interaction of this molecule with PD-L1 expressed on tumor cells. Furthermore, several studies introduce genes into CAR-T cells encoding blocking anti-PD1, anti-PD1 and anti-CTLA-4, or anti-PD-L1 antibodies, which after secretion by the CAR-T cells result in the same prevention of inhibition by the tumor cells (Figure 7). One study introduced a gene encoding an anti-PD1 nanobody with the same purpose (not shown). Moreover, some trials combined CAR-T cell therapy with anti-PD1, anti-CTLA-4 antibodies ([197,199], NCT03980288, NCT03726515, NCT01822652, NCT04003649 (see Table S1)).

As already described above, the use of allogeneic CAR-T cells has certain advantages. Therefore, several trials use T cells that are genetically modified in such a way that they are not recognized by the endogenous immune system, or can harm healthy tissue of the patient by knocking out the endogenous TCR and/or β2M, the latter resulting in the absence of MHC class I expression on the cell surface (Figure 7). This approach can lead to an off-the-shelf therapy using allogeneic T cells for many different patients.

## 4. CAR-T Cell Clinical Trials against Solid Tumors—Patient Pretreatments, Injection Sites, Safety Measurements, Clinical Outcomes

### 4.1. Treatments of Patients before CAR-T-Cell Transfer

To provide the best conditions for the introduced CAR-T cells, it is common to perform a lymphodepleting pretreatment of the patients. This is based on results obtained after transfer of tumor infiltrating lymphocytes (TILs) and CD19-directed CAR-T cells. In these studies, it was shown that lymphodepleting or conditioning chemotherapy administered prior to T-cell infusion clearly improve persistence and efficacy of these T cells [200], for example by reducing the number of suppressive cells, or removing competing sink cells, making IL-7 and IL-15 cytokines available for T cell expansion [201].

**Figure 8:**
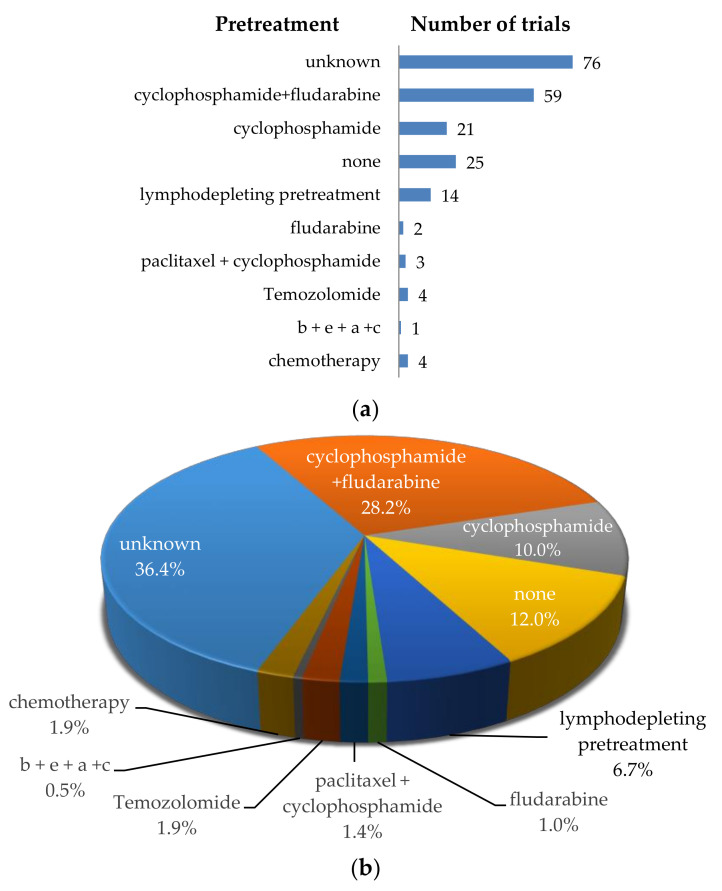
Schematic overview of the pretreatments used before CAR-T cells were applied in patients. (**a**) Number of clinical trials using a specific pretreatment; (**b**) Proportional distribution of clinical trials per pretreatment. Data was extracted from clinicaltrials.gov.

Different types of lymphodepleting or conditioning chemotherapies were also performed in the CAR-T-cell clinical trials against solid tumors (Figure 8). Mostly, the classical non-myeloablative lymphodepleting regimen with cyclophosphamide and fludarabine [202] was performed (*n* = 59, 28.2%; Figure 8), but also schedules with cyclophosphamide or fludarabine as single agent are described (*n* = 21 and *n* = 2, respectively; Figure 8). Other chemotherapies include: paclitaxel + cyclophosphamide, Temozolomide [203], or bis-1-nitrosourea + etoposide + arabinoside + cyclophosphamide (b + e + a + c) (Figure 8). A total of 93 trials do not clearly state if preconditioning is performed (unknown, *n* = 75), or what kind of preconditioning is performed (lymphodepleting pretreatment, *n* = 14; chemotherapy, *n* = 4) (Figure 8). In the registered clinical trials that describe the timing of lymphodepletion, the interval between lymphodepletion and CAR-T-cell application is mostly performed 3–5 days before the infusion of CAR-T cells, for 2–4 days. Twenty-five trials explicitly mention that no lymphodepletion is executed (Figure 8).

### 4.2. Injection Sites for CAR-T Cell Application

**Figure 9:**
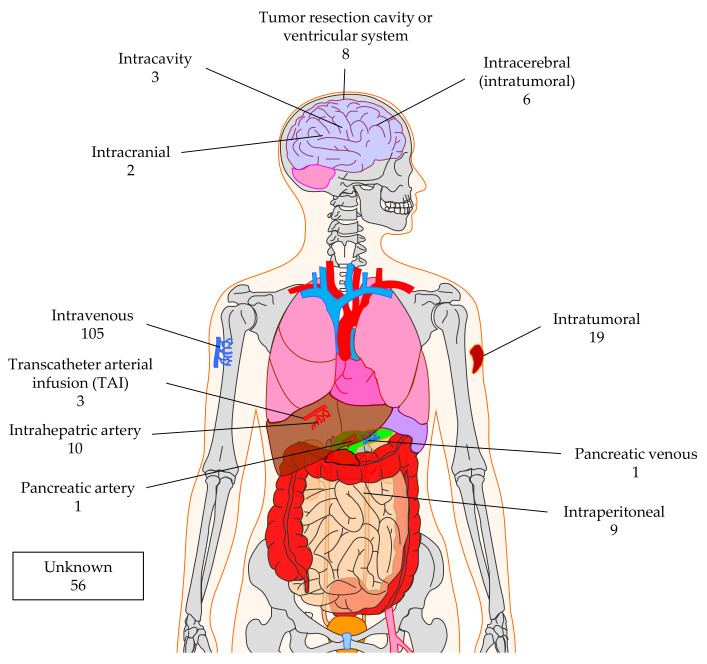
Schematic overview of the injection sites used to apply CAR-T cells against solid tumors. The numbers indicate the number of clinical trials using this injection site. Data was extracted from clinicaltrials.gov. The Motifolio Scientific Illustration Toolkit was used for the generation of this figure.

Most clinical trials apply the CAR-T cells by injecting them intravenously (*n* = 105; Figure 9) counting on the correct homing of the T cells to the tumor. However, there are also other sites possible, especially if one wants to apply the CAR-T cells very locally in the tumor or at the resection site. For example, trials treating brain tumors use intracranial (*n* = 2), intracavity (*n* = 3), or intracerebral (*n* = 6) injection, or inject into the ventricular system (*n* = 8) (Figure 9). Nineteen clinical trials indicated intratumoral injection, and nine use intraperitoneal injection (Figure 9). Local treatment of liver or pancreas cancers with CAR-T cells can be achieved by transcatheter arterial infusion (TAI; *n* = 3), intrahepatic artery injection (*n* = 10), pancreatic artery (*n* = 1), or pancreatic venous (*n* = 1) injection (Figure 9). A total of 56 clinical trials do not indicate the site of CAR-T-cell application (Figure 9).

### 4.3. Safety Measurements to Control Negative Effects of CAR-T Cells in the Patient

**Figure 10:**
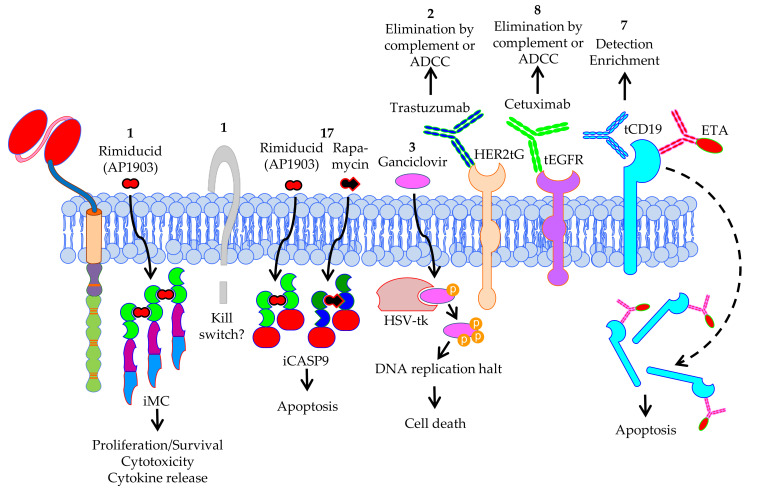
Schematic overview of safety measurements used in the treatment with CAR-T cells against solid tumors. The numbers indicate the number of clinical trials using this safety measurement. Data was extracted from clinicaltrials.gov. The Motifolio Scientific Illustration Toolkit was used for the generation of this figure.

As already mentioned above, most antigens targeted in clinical trials with CAR-T cells against solid tumors are not perfectly tumor specific. It can be that the antigens are expressed to some extend on normal healthy tissues, and there an on-target/off-tumor toxicity due to the accidental killing of non-malignant bystander cells co-expressing the target antigen can be induced by the CAR-T cells [11]. To be able to shut-off the CAR-T cells as soon as toxicity is noticed in the patient, several strategies were developed (Figure 10). Rimiducid (AP1903) and rapamycin are molecules that are able to induce dimerization of constructs containing inducible caspase 9, which are co-introduced with the CAR into the T cells as a suicide switch. After dimerization, the caspase 9 induces apoptosis of the CAR-T cells and thereby the unwanted/unexpected T-cell activities are eliminated [204,205]. This kind of suicide switch is used in 17 clinical trials (Figure 10) performed with mostly fourth generation safety CAR-T cells (4SCART). Interestingly, rimiducid (AP1903) is also used to provide multimerization of an inducible co-stimulatory molecule based on MyD88 and CD40 (iMC) into T cells, which allows for the selective activation of adoptively transferred T cells in vivo resulting in enhanced anti-tumor activity in solid tumors (Figure 10). Removal of rimiducid will switch off this co-stimulation again [206,207].

One of the oldest suicide switches used is the herpes simplex virus-thymidine kinase/ganciclovir (HSV-tk/GCV) strategy. Mechanistically, HSV-tk phosphorylates GCV and the resulting triphosphate form is incorporated by DNA polymerases into the DNA, leading to chain termination and cell death [208]. HSV-tk/GCV also induces apoptosis [209]. A disadvantage of using HSV-tk/GCV is that it can be immunogenic in immunocompetent patients causing a limited persistence of HSV-tk transduced cells [210]. Nevertheless, three clinical trials still use the HSV-tk/GCV strategy (Figure 10) [211].

Furthermore, several trimmed molecules are used for selection and/or depletion of CAR-T cells, like truncated HER2 (HER2tG), truncated EGFR (tEGFR), and truncated CD19 (tCD19) (Figure 10). Trastuzumab (Herceptin^®^) binds to HER2tG [212] and is used in two clinical trials for the elimination via complement or antibody-dependent cell-mediated cytotoxicity (ADCC) of CAR-T cells in case of on-target/off-tumor reactions (Figure 10). Cetuximab is used for the ablation of tEGFR-expressing CAR-T cells in eight clinical trials (Figure 10) via the same mechanisms [213]. Seven clinical trials use truncated CD19 either as selection marker for CAR-positive T cells [214], or as marker for elimination using an anti-CD19 antibody conjugated to pseudomonas toxin (CD19-ETA’) [215] (Figure 10). One clinical trial is using an undisclosed ‘kill switch’ as safety measurement (Figure 10).

**Figure 11:**
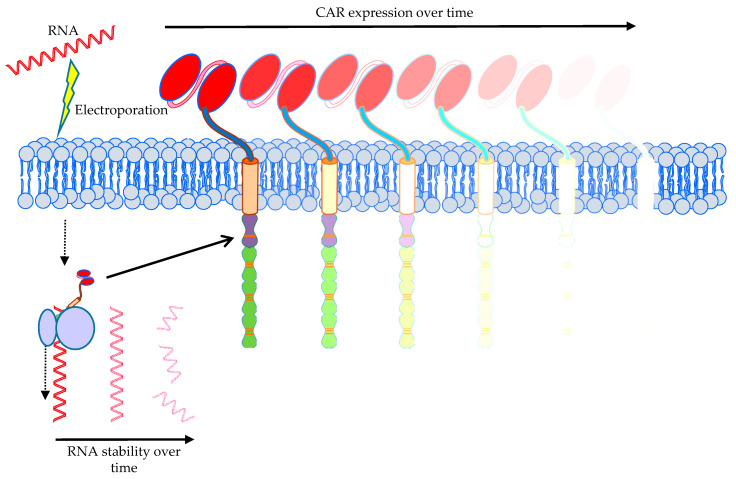
Schematic representation of introduction of a CAR by mRNA electroporation. Indicated are the low stability of the introduced mRNA over time and the transient expression of the CAR on the T-cell surface. The Motifolio Scientific Illustration Toolkit was used for the generation of this figure.

A special safety measurement to circumvent prolonged autoimmunity induced by an on-target/off-tumor reaction of the CAR is the introduction of the CAR by mRNA electroporation (*n* = 5, 2.6%; Figure 4). We have previously demonstrated that transient transfection of T cells with CARs using mRNA electroporation might be an effective and safe tool in cancer immunotherapy [121,216,217,218,219,220]. The electroporation procedure is based on complex physicochemical mechanisms leading to plasma membrane perforation upon application of electric fields allowing for subsequent entry of mRNA into the cytosol [221]. Using RNA-transfected CAR-T cells offers the advantage that the receptor expression is temporally restricted (Figure 11), rendering potential off-target and on-target/off-tumor toxicity transient as well. The CAR-RNA transfer strategy is especially attractive in phase 0/1 clinical trials exploring new tumor antigens for CAR-T-cell therapy with an unknown clinical safety profile.

The mRNA transfection strategy for CARs proposed by us quite some time ago [109] has in the meantime been applied by others in clinical trials. In patients with solid tumors c-MET was used as a CAR-target antigen on breast cancer and melanoma [222], (NCT01837602; NCT03060356) and mesothelin as a CAR-target antigen on mesothelioma, pancreatic cancer, and ovarian cancer [223,224,225], (NCT03608618; NCT01897415; NCT01355965). RNA transfection was even explored with non-solid tumors using CD19 and CD123 as target antigen [226], (NCT02277522; NCT02624258; NCT02623582). The mRNA-CAR-T cells in these studies were well tolerated [222], the cells migrated to primary and metastatic tumor sites, showed a clinical anti-tumor activity, and showed no evidence of on-target/off-tumor toxicity against normal tissues [223]. After local application, c-MET-CAR-T cells induced necrosis within the tumor. Importantly, some of the injected c-MET-CAR-T cells entered the blood stream and could be monitored in the circulation for a short time [222].

The clinical trials published by Beatty et al. and Maus et al., using mesothelin as antigen, showed a cytokine release syndrome (CRS) in one mesothelioma patient resulting in adverse events (anaphylaxis, cardiac arrest, respiratory failure, disseminated intravenous coagulation) within minutes of completing the third infusion [223,225]. In contrast, in pancreatic cancer patients no cytokine release syndrome and no dose-limiting toxicities, but actually stable disease in two of six patients were seen [224]. When using RNA-CAR-T cells, robust proliferation and persistence are not so important, making lymphodepletion unnecessary, as the transient receptor expression per se necessitates repetitive injections. Unlike most of the trials registered in clinicaltrials.gov which use virally transduced cells, which have to be applied only once, presuming that these cells will proliferate upon tumor-antigen recognition, making repeated applications unnecessary, RNA-transfected cells will lose CAR expression (Figure 11) and have to be replenished from the outside to maintain cytolytic pressure on the tumor.

The possible reason for the severe adverse events in the patient described above by Maus et al. and Beatty et al. [223,225], was that the CAR was based on a murine antibody and the adverse event was caused by IgE antibodies specific for the scFv in the CAR (i.e., a human anti-mouse antibody (HAMA) response), subsequently causing a CRS after an additional injection of CAR-T cells. These antibodies were probably induced by the intermittent dosing schedule of the CAR-T cells [223,225]. After the first two injections of RNA CAR-T cells on day 0 and day 7, the third injection was given after a long waiting period on day 49. This is sufficient time to complete an isotype switch from IgG to IgE. Therefore, rapid repetition of infusions seems to be best to prevent isotype switching if a HAMA response is induced.

Although the transient expression of a CAR on the cell surface of T cells by electroporation with mRNA can be an advantage if an on-target/off-tumor response is induced by the CAR-T cells, it can also be a disadvantage for the applicability. It has to be carefully monitored whether the infused CAR-T cells reach their tumor target in time before the CAR expression is too low for an effective anti-tumor response. This might be circumvented by local infusion of these CAR-T cells at the tumor site, as it is performed in several clinical trials (NCT01355965 [223,225], NCT01897415 [224], NCT03608618, NCT01837602 [222]). Furthermore, the necessary repetitive application of mRNA-transfected CAR-T cells harbors some hazards, like elaborately described above (e.g., possible isotype switch of a HAMA response [223,225]). Additionally, this repetitive application necessitates the production and storage of several batches of CAR-T cells, which might be cumbersome. Moreover, an anti-tumor CAR-T-cell memory will not be induced in patients treated with mRNA-transfected CAR-T cells, which might be a problem if the tumor is not completely eradicated and can reoccur. To draw final conclusions on the applicability of mRNA-transfected CAR-T cells, analysis in several clinical trials is necessary. To mitigate safety concerns, another promising strategy is the initial use of repetitive injections of RNA-transfected CAR-T cells to probe for toxicity, and in the case of no serious side-effects, switch to permanently transfected CAR-T cells.

### 4.4. Clinical Outcomes and Adverse Events of CAR-T-Cell Therapy of Solid Tumors

#### 4.4.1. Clinical Outcomes

Of 42 clinical trials using CAR-T cells against solid tumors registered at clinicaltrials.gov the clinical outcome could be retrieved from either clinicaltrials.gov, or through literature search on pubmed.ncbi.nlm.nih.gov (see Table S1; also including data on number of injected cells, trial phase, (estimated) patient number, trial status, principle investigator, and references) [25,197,199,227,228,229,230,231,232,233,234,235,236,237,238,239,240,241,242,243,244,245,246,247]. Some clinical outcomes were found in abstracts of ASCO meetings published in the Journal of Clinical Oncology (Table S1). Of the 375 treated patients listed in publications reporting on clinical outcome, 13 had a complete response, 35 had a partial response, 4 had a mixed response, 121 had a stable disease, 109 had a progressive disease, 8 had no evidence of disease, 5 were not evaluable, and of 80 patients the clinical outcome was not disclosed. This data is summarized in Table S2.

#### 4.4.2. Adverse Events

**Figure 12:**
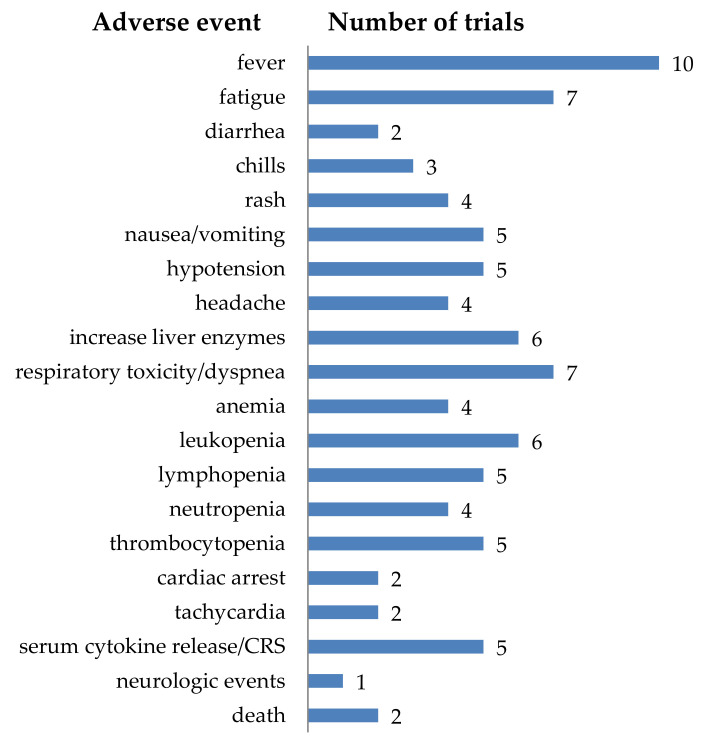
Schematic overview of the adverse events (all grades) described during the treatment of solid tumors with CAR-T cells. The number of clinical trials reporting a specific adverse event is indicated. Data was extracted from clinicaltrials.gov and literature search on pubmed.ncbi.nlm.nih.gov.

In total, 28 clinical trials described in this review also reported on adverse events (Table S1). The adverse events were quite diverse (Figure 12). Some adverse events were very local, and this could be explained by looking at the tumor site (e.g., seizure when treating glioblastoma, or abdominal pain when treating tumors in the liver). However, there were also more general adverse events, for example: fever, fatigue, nausea/vomiting, respirator toxicity/dyspnea, etc. (Figure 12). Although only five clinical trials directly reported on serum cytokine release or cytokine release syndrome (CRS) (Figure 12), this is probably an underestimation. For acute lymphoblastic leukemia (ALL) it is described that in 77% of the patients treated with CD19-CAR-T cells, CRS is prevalent [6]. It is reported that in these patients the clinical manifestations of CRS include a plethora of symptoms including mild fever with headache and myalgia, but also high fever, hypotension, acute respiratory distress syndrome, disseminated intravascular coagulation, organ failure, and death. Furthermore, elevated values for C-reactive protein (CRP) and IL-6, and signs of multi-organ failure, deranged coagulation parameters, and cytopenias are described [248]. The listed adverse events in Figure 12, and the data on all reported adverse events summarized in detail in Table S1, together with the description of the symptoms of CRS in ALL patients, suggests that more cases of cytokine release syndrome were induced by the treatment of solid tumors with CAR-T cells.

Another adverse event described for CAR-T-cell therapy is CAR-induced neurotoxicity [249], however, the mechanism of this neurotoxicity is not clear yet. Symptoms of neurotoxicity include transient cognitive impairments, hallucinations, and delirium, but also encephalopathy and seizures [249]. The above described seizure in the treatment of glioblastoma patients (NCT02209376; Table S1) might therefore also be a sign for neurotoxicity. Furthermore, one trial describes neurologic events (NCT00730613; Table S1), and in another trial one of the adverse events were olfactory auras (NCT02208362; Table S1), which both might indicate neurotoxicity.

## 5. Conclusions

This summarizing review on all the clinical trials using CAR-T cells against solid tumors registered at clinicaltrials.gov shows that many strategies are followed using many different CAR formats, application routes, and extra features introduced into the T cells. This probably indicates that the ideal strategy for treating solid tumors with CAR-T cells has not been found yet. This can also be seen in the clinical outcomes of the trials that reported on this; only 52 of 375 patients responded. Notwithstanding, the use of CAR-T cells in the treatment of solid tumors bears great opportunities, and further development and clinical testing is necessary to be able to respond to the high medical need for a treatment of such cancers.

Future clinical trials should be focused on testing new CAR formats. This not only includes testing new extracellular antigen-binding domains, but also formats increasing the safety of CAR-T-cell usage (e.g., bi-specific CARs, or split CARs) [250], and new intracellular signaling domains [251]. Furthermore, new vehicles for CARs [251,252] hold great promise for broadening the applicability. For example, the possibility of the off-the-shelf use of CAR-NK cells [253] or allogeneic CAR-T cells [254] can reduce costs for CAR-cell therapy and make it affordable for many more patients.

Furthermore, antigens which are more tumor specific should be found to prevent on-target/off-tumor reactions. Promising in this area are antigens expressed on the tumor stroma, which can also be targeted by CAR-T cells [255]. Targeting multiple antigens by one CAR-T cell (i.e., expression of different CARs specific for different antigens on one cell) can increase the tumor specificity and lessen the risk of off-target effects, and when intracellular signaling modules are split between the CARs, this can even increase the safety profile of the CAR-T cells. Additionally, the generation of antigen-loss variants of the tumors is less likely.

Moreover, combination therapies of CAR-T cells with various small molecules and monoclonal antibodies to circumvent tumor escape and increase anti-tumor activity are already clinically tested in many hematologic tumors (reviewed in detail in [171]). Such combinations also hold great promise for the treatment of solid tumors and need to be tested in clinical trials in the near future.

## Figures and Tables

**Table 1:** Summary of antigens expressed by solid tumors in different organs targeted by. CAR-T cells in trials registered at clinicaltrials.gov.

Antigen	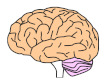	Several Organs	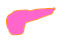	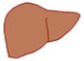	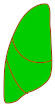	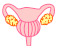	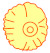	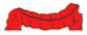	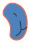	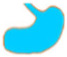	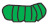	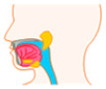	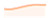	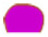	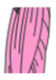	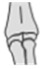	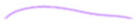	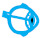	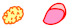	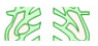	Number of Trials
AFP peptide/A2				√																	**2**
B7-H3	√	√		√					√				√		√	√		√	√	√	**6**
CD20													√								**1**
CD44v6							√			√	√										**2**
CD70			√			√	√		√				√								**2**
CD133	√		√	√		√	√	√													**3**
CD147 (EMMPRIN)	√			√																	**2**
CD171 (L1CAM)									√												**2**
CEA		√	√	√	√	√	√	√		√							√				**16**
claudin 18.2 (CLD18)		√	√							√											**6**
c-Met/hepatocyte growth factor receptor							√						√								**4**
DLL3 (delta-like protein 3)					√																**1**
EGFR	√	√	√	√	√	√		√	√	√					√	√		√	√	√	**8**
EGFR family member		√																			**1**
EGFRvIII	√																				**11**
EGFR806	√																				**1**
EpCAM			√	√			√	√		√	√	√					√				**6**
EphA2	√																				**2**
ErbB2 dimers												√									**1**
FAP					√																**1**
FBP (folate binding protein)						√															**3**
GD2	√	√							√				√			√		√			**24**
gp100 (209-217/HLA-A2)													√								**1**
GPC3 (glypican-3)		√		√	√				√						√				√		**18**
HER2	√	√	√	√	√	√	√	√		√		√		√							**17**
ICAM1												√									**1**
IL13Rα2	√												√								**6**
Lewis Y		√																			**2**
ligands of chlorotoxin	√																				**1**
LMP1 (EBV)												√									**1**
mesothelin		√	√		√	√	√										√				**32**
MG7				√																	**1**
MUC1	√	√	√	√	√		√	√		√		√									**11**
Muc1 (cleaved form)							√														**1**
MUC16ecto		√				√											√				**1**
TnMuc1		√	√		√	√	√														**1**
Nectin4/FAP		√	√		√	√	√							√							**1**
NKG2D-ligands (MIC-A,-B, ULBP-1,-2,-3,-4,-5,-6)	√	√	√	√		√	√	√		√	√	√		√							**6**
PD-L1	√				√																**5**
PSCA		√	√							√	√										**4**
PSMA	√	√							√		√										**11**
ROR1		√			√		√														**1**
ROR2		√	√							√				√	√						**2**
VEGFR2									√				√								**1**
Several Ags	√	√	√	√	√	√	√	√	√	√		√	√	√		√					**16**
undisclosed antigen						√											√				**2**

**Table 2:** Summary of antigens targeted on different tumors by CAR-T cells in trials registered at clinicaltrials.gov.

Organ	Cancer Type	Targeted Antigens
brain/CNS	brain	CD133, HER2, PSMA
	glioma	B7-H3, CD147, EGFR, EGFRvIII, EphA2, GD2, HER2, IL13Rα2, MUC1, CD133
	glioblastoma	B7-H3, ligands of chlorotoxin, EGFRvIII, HER2, IL13Rα2, NKG2D-Ligands, PD-L1
	primitive neuroectodermal tumor	B7-H3
	choroid plexus carcinoma	B7-H3
	pineoblastoma	B7-H3
	CNS tumor	B7-H3, EGFR806, HER2
	ependymoma	B7-H3
	medulloblastoma	B7-H3, NKG2D-Ligands
several organs	rhabdoid tumor	B7-H3, EGFR, GPC3
	Rhabdomyosarcoma	B7-H3, EGFR, GPC3
	desmoplastic small round cell tumor	B7-H3, EGFR
	sarcoma	GD2, HER2, NKG2D-Ligands, CD133, MUC1, CD117
	adenocarcinoma	CEA
	solid tumors	B7-H3, CEA, claudin 18.2, EGFR, EGFR family member, GD2, GPC3, HER2, Lewis Y, mesothelin, MUC1, MUC16ecto, TnMuc1, Nectin4, ROR2
pancreas	pancreatic	CD70, CD133, CEA, claudin 18.2, EGFR, EpCAM, HER2, mesothelin, MUC1, Nectin4, NKG2D-Ligands, PSCA, ROR2, EGFRvIII
	pancreatic ductal adenocarcinoma	claudin 18.2, mesothelin, TnMuc1
liver	liver	CD133, CEA, EGFR, EpCAM, GPC3, MG7, NKG2D-Ligands
	HCC (hepatocellular carcinoma)	AFP/HLA-A2, CD147, GPC3, MUC1, NKG2D-Ligands, c-MET, PD-L1
	hepatoblastoma	B7-H3, EGFR
	hepatoma	several
	gall bladder carcinoma	EGFR
	cholangiocarcinoma	EGFR, HER2, MUC1
lung	lung	CEA, EGFR, HER2, mesothelin, Lewis Y, PSCA, MUC1, PD-L1, CD80/86, MAGE-A1, MAGE-A4, GD2
	small cell lung cancer	DLL3
	mesothelioma	FAP, mesothelin
	lung squamous cell carcinoma	GPC3
	NSCLC	EGFR, mesothelin, MUC1, TnMuc1, Nectin4, PD-L1, ROR1, CD80/86
uterus/cervix	ovarian	CD70, CD133, CEA, EGFR, FBP, HER2, mesothelin, TnMuc1, Nectin4, NKG2D-Ligands
	cervical	mesothelin, GD2, PSMA, MUC1, mesothelin
	fallopian tube	mesothelin, TnMuc1
breast	breast	CD44v6, CD70, CD133, CEA, c-MET, EpCAM, HER2, mesothelin, Muc1 (cleaved from), Nectin4, GD2
	TNBC	c-MET, mesothelin, MUC1, TnMuc1, NKG2D-Ligands, ROR1
colon	colorectal	CD133, CEA, EGFR, HER2, MUC1, NKG2D-Ligands
	colon	EpCAM, HER2, NKG2D-Ligands
kidney	renal	CD70, EGFR, VEGFR2, ROR2, AXL
	neuroblastoma	B7-H3, CD171, EGFR, GD2, PSMA
	wilms tumor	B7-H3, EGFR, GPC3
stomach	gastric	CD44v6, CEA, claudin 18.2, EGFR, EpCAM, HER2, MUC1, NKG2D-Ligands, PSCA, ROR2
prostate	prostate	CD44v6, EpCAM, NKG2D-Ligands, PSCA, PSMA
head/neck	esophageal	EpCAM, HER2, MUC1
	nasopharyngeal	EpCAM, LMP1, NKG2D-Ligands
	SCCHN	ErbB dimers, HER2
	salivary gland	HER2
	thyroid cancer	ICAM1
skin	melanoma	B7-H3, CD20, CD70, c-MET, GD2, gp100/HLA-A2, IL13Rα2, VEGFR2
bladder	bladder	HER2, Nectin4, NKG2D-Ligands, ROR2, PSMA, FBP
soft tissue	synovial sarcoma	B7-H3, EGFR
	clear cell sarcoma	B7-H3, EGFR
	soft tissue sarcoma	B7-H3, EGFR, GPC3, ROR2
bone	osteosarcoma	B7-H3, EGFR, GD2
	ewing sarcoma	B7-H3, EGFR
abdomen	peritoneal	CEA, EpCAM, mesothelin
eye	retinoblastoma	B7-H3, EGFR
	uveal melanoma	GD2
ovary/testis	germ cell tumor	B7-H3, EGFR, GPC3
peripheral nerves	malignant peripheral nerve sheath tumor	B7-H3, EGFR

## Supplementary Materials

The following are available online at https://www.mdpi.com/2072-6694/12/9/2567/s1, Table S1: Characteristics of all clinical trials using CAR-T cells against solid tumors. Data was collected from clinicaltrials.gov and literature search on pubmed.ncbi.nlm.nih.gov; Table S2: Summary of clinical trials reporting on clinical outcome. Data was collected from clinicaltrials.gov and literature search on pubmed.ncbi.nlm.nih.gov.
