# Supplementary material for: The Landscape of CAR-T Cell Clinical Trials against Solid Tumors—A Comprehensive Overview
Source: Cancers (Basel). 2020 Sep 9;12(9):2567. doi: 10.3390/cancers12092567 (PMC7563774; doi:10.3390/cancers12092567)
Supplement: Supplementary file 1 [file cancers-12-02567-s001.zip › Supplementary Table S2.pdf]

Supplemental Table S2: Summary of clinical trials reporting on clinical outcome. Data was collected from clinicaltrials.gov and literature search on pubmed.ncbi.nlm.nih.gov.

**NCT**	**# patients**	**CR**	**PR**	**SD**	**PD**	**NE**	**NED**	**MR**	**undisclosed**
NCT03349255	6	1	2						3
NCT02541370	23		3	14					6
NCT00006480	6		1		5				
NCT01373047	6			1	5				
NCT02349724	10			7	2	1			
NCT02416466	6			3					3
NCT01212887	14			7	7				
NCT03159819	12	1	3	5					3
NCT01837602	6			1	2				3
NCT01869166	28	1	2	15	4				6
NCT02209376	10			1	2				7
NCT01818323	15	1		9					5
NCT02107963	4		4						
NCT00085930	19	3	1	1	6		8		
NCT01460901	3		3						
NCT01822652	11			3	8				
NCT03146234	5		1		1	1			2
NCT02395250	8		1	2	2	3			
NCT01935843	11		1	5	5				
NCT01109095	16		1	7	8				
NCT00902044	27	2	1	7	12				5
NCT03054298	1	1							
NCT01583686	15			1	14				
NCT02159716	6			6					
NCT02414269	14	2	5	4					3
NCT01355965	6	1		4	1				
NCT01897415	16			2					14
NCT02587689	1		1						
NCT03310008	3		1						2
NCT03018405	14			4					10
NCT02744287	15			8	3				4
NCT01929239	5		2						3
NCT02706392	9		1	3				4	1
NCT01218867	24		1	1	22				
	**375**	**13**	**35**	**121**	**109**	**5**	**8**	**4**	**80**

CR=complete response, PR=partial response, SD=stable disease, PD=progressive disease, NE=not evaluable, NED=no evidence of disease, MR=mixed response.
