# Supplementary material for: The Landscape of CAR-T Cell Clinical Trials against Solid Tumors—A Comprehensive Overview
Source: Cancers (Basel). 2020 Sep 9;12(9):2567. doi: 10.3390/cancers12092567 (PMC7563774; doi:10.3390/cancers12092567)
Supplement: Supplementary file 1 [file cancers-12-02567-s001.zip › Supplementary Table S1.pdf]

Supplemental Table S1: Characteristics of all clinical trials using CAR-T cells against solid tumors. Data was collected from clinicaltrials.gov and literature search on pubmed.ncbi.nlm.nih.gov.

Antigen	Cancer	CAR type /add-ons	CAR transfer	Mode of injection	Number of cells	Phase	Patient # /pretreatment /other drugs	Outcome	NCT# /Status /PI	Ref	Origin	Adverse events
AFP (alpha feto-protein)/HLA-A2	HCC	*ET1402L1-CART<br>*Human antibody domain against the anti-HLA-A02/ AFP complex<br>*scFv-CD28/CD3ζ	lentiviral	iv, iha	dose escalation	phase 1	*est. enrollment: 18	1/6 CR, 2/6 PR	NCT03349255; Terminated (Will study new T-cell construct for the same indication); PI: Qibin Song	[158]	Renmin Hospital of Wuhan University, China	
AFPpeptide/HLA-A2	*HCC<br>*(metastatic) Liver cancer	*2nd gen. ARTEMIS™ receptor engineered with a human antibody domain against the anti-HLA-A02/AFP complex	lentiviral	iv, it, iha	dose escalation	early phase 1	*est. enrollment: 27		NCT03888859; recruiting<br>PI: Chang Liu		First Affiliated Hospital Xi'an Jiaotong University, China	
B7-H3	*Recurrent glioblastoma<br>*Refractory glioblastoma	?	retroviral	it, intracere-broventricular	?	phase 1/2	*est. enrollment: 40<br>*Temozolomide		NCT04077866; recruiting;<br>PI: ?		*Second Affiliated Hospital of Zhejiang University<br>*School of Medicine, Hangzhou, Zhejiang, China<br>*Huzhou Central Hospital, Huzhou, Zhejiang, China<br>*Ningbo Yinzhou People's Hospital, Ningbo, Zhejiang, China	
B7-H3	*Central nervous system tumor<br>*Diffuse intrinsic pontine glioma<br>*Diffuse midline glioma<br>*Ependymoma<br>*Medulloblastoma<br>*Childhood germ cell tumor<br>*Atypical teratoid/ rhabdoid tumor<br>*Primitive neuroectodermal tumor<br>*Choroid plexus carcinoma<br>*Pineoblastoma<br>*Childhood glioma	*B7H3-specific 2nd gen. CAR<br>*EGFRt	lentiviral	tumor resection cavity or ventricular system	?	phase 1	est. enrollment: 70		NCT04185038; recruiting;<br>PI: Nick Vitanza		Seattle Children's Hospital, Seattle, Washington, USA	
B7-H3	*Pediatric solid tumor<br>*Germ cell tumor<br>*Retinoblastoma<br>*Hepatoblastoma<br>*Wilms tumor<br>*Rhabdoid tumor<br>*Carcinoma<br>*Osteosarcoma<br>*Ewing sarcoma<br>*Rhabdomyosarcoma<br>*Synovial sarcoma<br>*Clear cell sarcoma<br>*Malignant peripheral nerve sheath tumors<br>*Desmoplastic small round cell tumor<br>*Soft tissue sarcoma<br>*Neuroblastoma<br>*Melanoma	Arm A:<br>*scFv-4-1BB/CD3ζ specific for B7-H3<br>*EGFRt for depletion with cetuximab<br>Arm B:<br>*scFv-4-1BB/CD3ζ specific for B7-H3 and<br>*scFv-4-1BB/CD3ζ specific for CD19<br>*Her2tG for depletion with trastuzumab	lentiviral	?	?	phase 1	est. enrollment: 68		NCT04483778; recruiting;<br>PI: Navin Pinto		Seattle Children's Hospital, USA	
B7-H3	r/r glioblastoma	?	retroviral	it, intracerebro-ventricular	3 infusions, 1-2 weeks interval	phase 1	*est. enrollment: 12<br>*Temozolomide		NCT04385173; recruiting;<br>PI:?		Second Affiliated Hospital, School of Medicine, Zhejiang University, China	
B7-H3 (CD276)	B7-H3 pos. solid tumor	4SCAR-276	lentiviral	?	approx. 10^6 CART cells/kg	phase 1/2	*est. enrollment: 100<br>*cy+flu		NCT04432649; recruiting;<br>PI: ?		*Sun Yat-sen University<br>*Shenzhen Children's Hospital<br>*Shenzhen Geno-Immune Medical Institute , China	
CD20	Unresectable stage III or IV melanoma	MB-CART20.1	lentiviral	iv	*MTD, 3+3 design with 1 log dose increments, maximum 3 dose levels<br>*level 1: 3+3 patients $1x10^5$ CART20.1/kg<br>*level 2: 3+3 patients $1x10^6$ CART20.1/kg<br>*level 3: 3+3 patients $1x10^7$ CART20.1/kg	phase 1	*est. enrollment: 15<br>*cy+flu		NCT03893019; recruiting;<br>PI: Peter Borchmann		*Miltenyi biotec; Bergisch Gladbach, Germany<br>* Universitätsklini-kum Köln, Germany	
CD44v6	Multiple cancers including - but not limited to -<br>*stomach cancer,<br>*breast cancer,<br>*prostate cancer	4SCAR	lentiviral	iv	10^6 cells/kg	phase 1/2	*est. enrollment: 100		NCT04427449; recruiting;<br>PI: ?		*Shenzhen Hospital of Southern Medical University<br>*The Seventh Affilliated Hospital, Sun Yat-Sen University<br>*Shenzhen Children's HospitalShenzhen Geno-Immune Medical Institute, China	

Antigen	Cancer	CAR type /add-ons	CAR transfer	Mode of injection	Number of cells	Phase	Patient # /pretreatment /other drugs	Outcome	NCT# /Status /PI	Ref	Origin	Adverse events
CD70	CD70-expressing cancers (e.g.<br>*renal carcinoma;<br>*pancreatic cancer,<br>*breast cancer,<br>*melanoma,<br>*ovarian cancer)	No scFv against CD70, but CD27 (i.e. CD70 ligand) as binding moiety	retroviral	iv	MTD	phase 1/2	*est. enrollment: 113<br>*cy+flu<br>*high dose IL-2 (Aldesleukin; 720,000IU/kg)		NCT02830724; recruiting;<br>PI: James Yang	[147,150]	NCI, NIH, USA	
CD70	Renal cell carcinoma	*Allogeneic CRISPR-Cas9-engineered T cells (CTX130)<br>*β2M knockout<br>*TRAC knockout by insertion of CAR gene (i.e. TCR knockout and CAR expression)	CRISPR-Cas9	iv	dose escalation	phase 1	*est. enrollment: 95<br>*lymphodepleting chemotherapy		NCT04438083; recruiting;<br>PI: Matthias Will		*CRISPR Therapeutics AG<br>*Research Site 1: Melbourne, Victoria, Australia	
CD133	Relapsed and/or chemotherapy refractory advanced malinancies<br>*liver,<br>*pancreatic,<br>*brain,<br>*breast,<br>*ovarian,<br>*colorectal cancer,<br>*acute myeloid and lymphoid leukemias	Comparison:<br>*scFv-4-1BB/CD3ζ<br>*scFv-CD3ζ	retroviral	iv	dose escalation, (3+3 approach)<br>Mean: 1.43x10⁶/kg	phase 1	*est. enrollment: 20	3/23 PR, 14/23 SD	NCT02541370; completed;<br>PI: Han Weidong	[39,227]	Chinese PLA General Hospital; China	*CD133: an intermittent upper abdominal dull pain, chills, fever, and rapidly deteriorative grade 3 systemic subcutaneous hemorrhages and congestive rashes together with serum cytokine release<br><br>*On-target/off-tumor toxicity: liver enzymes increase (aEGFR), rash and mucositis (aCD133)
CD147 (EMMPRIN (extracellular matrix metallopro-teinase inducer), Basigin)	Recurrent malignant glioma	?	?	ic	MTD, 3 doses, 1-week intervals, 3+3	early phase 1	*est. enrollment: 31		NCT04045847; recruiting;<br>PI: not provided		Xijing Hospital, China	
CD147 (EMMPRIN (extracellular matrix metallopro-teinase inducer), Basigin)	Very advanced HCC	?	?	iha	MTD, 3 doses, 1-week intervals	phase 1	est. enrollment: 34		NCT03993743; recruiting;<br>PI: ?		Fourth Military Medical University; Xijing Hospital, China	
CD171 (L1CAM)	Neuroblastoma	*scFv-4-1BB/CD3ζ<br>*scFv-CD28/4-1BB/CD3ζ<br>*EGFRt	lentiviral	iv	1x10⁶cells/kg,<br>5x10⁶ cells/kg,<br>1x10⁷ cells/kg,<br>5x10⁷ cells/kg,<br>1x10⁸ cells/kg	phase 1	*est. enrollment: 40<br>*lymphodepletion		NCT02311621; recruiting;<br>PI: Julie Park	[133]	Seattle Children's Hospital, USA	
CD171 (L1CAM)	Neuroblastoma	*CE7R CAR<br>*scFv-CD3ζ<br>*Genetically-modified autologous CD8+ T-cell clones<br>*HyTK	genetically modified?	iv	1x10⁸-1.1x10⁹/m² (split dose; 2-3 times infusions 14 days apart)	phase 1	*IL-2<br>*chemotherapy regimen that may consist of one of the following: cyclophosphamide and topotecan; ifosfamide, carboplatin, and etoposide; or another chemotherapy regimen	*1/6 PR and 5/6 PD 56 days after adoptive therapy<br>*But all patients died of disease ultimately with only one achieved a relatively long survival.	NCT00006480; completed;<br>PI: Julie Park	[211]	*Fred Hutchinson Cancer Research Center<br>*National Cancer Institute (NCI), USA	*Grade 3 lymphopenia, neutropenia, low hemoglobinand bacteremia caused by 10⁸/m² CAR-T cells<br>*Grade 3 pneumonitis for one patient, which was associated with 10⁹/m² CAR-T cells<br>*No grade 4 or 5 adverse event was found<br>*On-target/off-tumor toxicity: lymphopenia, neutropenia, anemia, pneumonitis
CEA	Pancreatic carcinoma with CEA+ liver metastases	CAR2 anti-CEA CAR	?	iha	*weekly 3 doses of CAR-T cells in each 28-day cycle, max 3 cycles<br>*1x10¹⁰ cell per dose	phase 1b	*est. enrollment: 6<br>*low dose systemic IL-2		NCT03818165; active, not recruiting;<br>PI: Steven C Katz		Roger Williams Medical Center, USA	
CEA	*Gastric adenocarcinoma<br>*colorectal adenocarcinoma<br>*lung adenocarcinoma<br>*Solid tumors	scFv-CD28/CD3ζ	retroviral	iv	?	phase 2	*est. enrollment: 48<br>*IL-2		NCT01723306; suspended (funding);<br>PI: Richard P Junghans		Roger Williams Medical Center, USA	
CEA	Adenocarcinomas	*scFv-CD3ζ<br>*murine scFv	?	?	dose escalation	phase 1	?		NCT00004178; completed;<br>PI: Richard P Junghans		*Beth Israel Deaconess Medical Center<br>*Roger Williams Medical Center, USA	
CEA	Metastatic breast cancer	scFv-CD28/CD3ζ	retroviral	iv	10⁹, 10¹⁰, 10¹¹	phase 1	*est. enrollment: 26<br>*with/without IL-2<br>*no preconditioning		NCT00673829; suspended (funding);<br>PI: Richard P Junghans		Roger Williams Medical Center, USA	
CEA	CEA+ liver metastases	*scFv-CD28/CD3ζ<br>*scFv based on hMN14 (humanized)<br>*CD8 hinge	retroviral	iha	*Intrapatient dose escalation<br>*10⁸, 10⁹, 10¹⁰<br>*Three infusions over the course of 6 weeks	phase 1	*actual enrollment: 8<br>*no preconditioning	*Katz et al., 2015:1/6 SD, 5/6 PD and DOD (however decrease in CEA and 4/6 had necrosis of liver lesions)<br>*Of 6 patients 3 got dose escalation 10⁸, 10⁹, 10¹⁰, without support; the other three got 3x10¹⁰ with systemic IL-2 support.<br>*Book chapter: no systemic toxicity; minority of patients showed increased survival or decreased serum CEA	NCT01373047; completed;<br>PI: Steven C Katz	[51-53]	Roger Williams Medical Center, USA	*Grade 3 fever and tachycardia (1 patient), associated to high-dose IL-2 administration.<br>*Grade 1/2 transient elevations of alkaline phosphatase, total bilirubin and aspartate aminotransferase levels observed in all patients.<br>*Mylagias, abdomnal pain, nausea, emesis, abdominal wall muscle spasm, ALT↑, AST↑, ascites, edema, thrombo-cytopenia, leukopenia, dyspnea, pleural effusion, anorexia, rash, subscapular liver hematoma,eosinophilia, chills, diarrhea, dehydration, colitis.<br>*On-target/off-tumor toxicity: no
CEA	*Pancreatic cancer<br>*liver metastases	?	?	iha		phase 2b	*est. enrollment: 167<br>*CAR-T w/ chemotherapy (gemcitabine/nab paclitaxel, NLIR+FU/FA, Capecitabine) vs. chemotherapy alone<br>* low dose IL-2		NCT04037241; not yet recruiting;<br>PI: not provided		Sorrento Therapeutics, Inc., USA	
CEA	Colorectal carcinoma	scFv-CD28/CD3ζ	retroviral	iv	10⁹, 10¹⁰, 10¹¹	phase 1	*actual enrollment: 1<br>*no preconditioning		NCT00673322,; terminated (this study will be incorporated into another study),;<br>PI: Richard P Junghans		Roger Williams Medical Center, USA	

Antigen	Cancer	CAR type /add-ons	CAR transfer	Mode of injection	Number of cells	Phase	Patient # /pretreatment /other drugs	Outcome	NCT# /Status /PI	Ref	Origin	Adverse events
CEA	Gastric cancer	scFv-CD28/CD3ζ	?	?	?	phase 1	actual enrollment: 0		NCT00429078; Withdrawn (low accrual); PI: Richard P Junghans		Roger Williams Medical Center, USA	
CEA	CEA-positve cancer: *lung, *colorectal, *gastric, *breast, *pancreatic cancer	*scFv-CD28/CD3ζ *scFv based on BW431/26 (humanized) *IgG4 hinge	lentiviral	iv	dose escalation, five escalating dose levels ($1x10^5$-$1x10^8$/CAR+ cells/kg), split doses (10, 30, and 60% of the total cell dose)	phase 1	*est. enrollment: 75 *cy+flu	*2/10PD, 7/10 SD, 1/10 not evaluated *Decline of serum CEA level in most patients *High dose well tolerated (no colitis, no respiratory toxicity)	NCT02349724; unknown; PI: Cheng Qian	[54]	Southwest Hospital, China	*Fever *On-target/off-tumor toxicity: no
CEA	CEA-expressing adenocarcinoma peritoneal metastases or malignant ascites: *colorectal, *gastric, *breast, *pancreas	?	?	ip	MTD, dose escalation one dose per patient	phase 1	est. enrollment: 18		NCT03682744; active, not recruiting; PI: Steven Katz		Roger WilliamsMedical Center, USA	
CEA	Liver metastases	scFv-CD28/CD3ζ	retroviral	iha in combination with Sir-spheres (SIRT)	fixed dose; 3 infusions over 6 weeks	phase 1b	*actual enrollment: 8 *no lymphodepletion *low dose IL-2	*Well tolerated *Biologic responses were demonstrated *AACR 2017: 3/6 SD, median OS 6.9 months, 1 metabolic CR	NCT02416466; completed; PI: Steven Katz	[55,56]	Roger Williams Medical Center, USA	On-target/off-tumor toxicity: no
CEA	*CEA-expressing liver metastases *Pancreas cancer	?	?	iha or pv	*Fixed dose *3 doses per patient with 1 week intervals	phase 1b	*actual enrollment: 5 *low dose IL-2	*n=5 (4 pancreatic cancer). 2 with no viable liver metastases by PET scan *Median OS post-treatment 8.3 months	NCT02850536; active, not recruiting; PI: Steven Katz and Richard Schulick	[57]	*Roger WilliamsMedical Center *University of Colorado, Denver, USA	
CEA	r/r CEA+ cancer: *solid tumor, *lung, *colorectal, *liver, *pancreatic, *gastric, *breast	?	?	iv	1-3 times	phase 1/2	est. enrollment: 40		NCT04348643 recruiting PI: Zhi Yang and Yingzi Zhang		Chongqing Precision Biotech Co., China	
CEA	*Breast, *colorectal, *gastric, *lung, *ovarian, *pancreatic cancer, *unspecified adult solid tumor	*scFv-CD3ζ *scFv based on MFE23 (murine)	retroviral	iv	dose escalation $1x10^9$-$5x10^{10}$	phase 1	*actual enrollment: 14 *Cohort 1-3: only flu *Cohort 4: cy+flu *Aldesleukin (IL-2)	*7/14 SD, 7/14 PD *Transient reduction in serum CEA in cohort 4, no colitis, transient, acute respiratory toxicity	NCT01212887; Terminated (due to safety concerns and lack of efficacy); PI: Robert E. Hawkins, MD	[58]	*Cancer Research UK *Christie Hospital Manchester, England, United Kingdom	*Pelvic pain, neutropaenia, general deterioration, hypocalcaemia, leukopenia, hypophosphataemia, lymphopenia, left side pain, abdominal pain, anaemia, thrombocytopenia, vomiting, hypotension, hyperbilirubinaemia, hyoialbuminaemia, epistaxis, hyponatraemia, intermittent pyrexia, haematemesis, jaundice, neutropenic sepsis, intermittent anaemia, intermittent increased respiratory rate *On-target/off-tumor toxicity: transient acute respiratory toxicity
claudin 18.2	Gastric cancer	LCAR-C18S cells	?	infusion?	*MTD/ RP2D regimen finding *single dose	phase 1	*est. enrollment: 34 *cy+flu		NCT04467853; not yet recruiting; PI: Jin Li		*Shanghai East Hospital *Nanjing Legend Biotech Co., China	
claudin 18.2	*Advanced gastric cancer *Pancreatic ductal adenocarcinoma	LCAR-C182A	?	iv	?	phase 1	*est. enrollment: 18 *cy+flu		NCT03890198; recruiting; PI: Enxiao Li		First Affiliated Hospital Xi'an Jiaotong University, China	
claudin 18.2 (CLD18)	*Advanced gastric adenocarcinoma *Pancreatic adenocarcinoma	scFv-CD28/CD3ζ	lentiviral	iv	dose escalation, multiple infusions	phase 1	*est. enrollment: 24 *Lymphodepletion	1/12 CR, 3/12 PR, 5/12 SD	NCT03159819; recruiting; PI: Xianbao Zhan	[228]	Shanghai hospital, China	
claudin 18.2	Advanced solid tumors; claudin 18.2 positive	humanized CAR	?	?	MTD	phase 1	*est. enrollment: 15 *cy+flu		NCT03874897; recruiting; PI: Lin Shen		Beijing Cancer Hospital, China	
claudin 18.2	*Gastric adenocarcinoma *Pancreatic adenocarcinoma	CT041	?	?	Part A: dose escalation (3+3) Part B: expansion cohort	phase 1b	*est. enrollment: 30 *Preconditioning		NCT04404595; not yet recruiting; PI: ?		Carsgen Therapeutics, Ltd., USA	
Ligand(s) of chlorotoxin	*Recurrent glioblastoma *Recurrent malignant glioma *Recurrent WHO grade II glioma *Recurrent WHO grade III glioma	*Chlorotoxin (EQ)-CD28/CD3ζ (no scFv, peptide-bearing CAR, with chlorotoxin peptide extracellularly) *CD19t	?	infusion?	Patients receive CAR-T lymphocytes via dual delivery starting on day 0 for 3 weekly cycles over 21 days. Each treatment cycle begins with one or two CAR-T cell infusions (one at each catheter site) and lasts for 1 week. Treatment continues in the absence of disease progression or unacceptable toxicity.	phase 1	est. enrollment: 36		NCT04214392; recruiting; PI: Behnam Badie	[147]	*City of Hope Medical Center; *National Cancer Institute (NCI), USA	
c-Met/hepatocyte growth factor receptor	*Metastatic breast cancer *TNBC	*scFv-4-1BB/CD3ζ *scFv based on Onartuzumab	mRNA-transfection	it (cutaneous or lymph node mets)	1x $3x10^7$ or 1x $3x10^8$	phase 1	*n=6 (3 each cohort) *no pretreatment	*mRNA c-Met-CAR-T cell injections were well tolerated *No drug-related adverse effects greater than grade 1 *Extensive tumor necrosis at injection site *Loss of c-Met immunoreactivity *Surrounded by macrophages at the leading edges and within necrotic zones *2/6 PD, 1/6 SD, 3/6 died	NCT01837602; completed; PI: Tchou	[222]	Upenn, USA	*No side effects *On-target/off-tumor toxicity: no

Antigen	Cancer	CAR type /add-ons	CAR transfer	Mode of injection	Number of cells	Phase	Patient # /pretreatment /other drugs	Outcome	NCT# /Status /PI	Ref	Origin	Adverse events
c-Met/hepatocyte growth factor receptor	*Advanced breast carcinoma<br>*Advanced melanoma	scFv-4-1BB/CD3ζ	mRNA-transfection	iv	*Up to 6 doses over a 2 week period<br>*Each dose is $1x10^8$ cells	early phase 1	*est. enrollment: 10 patients<br>*no lymphodepletion		NCT03060356; terminated (halt in funding);<br>PI: Mitchell		Upenn, USA	
DLL3 (delta-like protein 3)	Relapsed/refractory small cell lung cancer (SCLC)	AMG 119	?	iv	single dose	phase 1	est. enrollment: 41		NCT03392064; active, not recruiting;<br>PI: MD Amgen		Amgen, USA	
EGFR	*Non-small cell lung cancer,<br>*colorectal cancer with liver metastasis<br>*Chemotherapy resistant or relapsed:<br>*ovary cancer,<br>*lung cancer,<br>*cholangiocarcinoma,<br>*gall bladder carcinoma,<br>*pancreatic cancer,<br>*renal carcinoma and<br>*other relapsed/ metastatic/ unresectable tumors	*scFv-4-1BB/CD3ζ<br>*scFv based on E10	lentiviral	iv	*1 to 3 cycles of CAR-T-EGFR<br>*Median CAR-T cell dose $2.65x10^6$/kg in Guo<br>*Median CAR-T cell dose $0.97x10^7$/kg in Feng<br>*Dose escalation over several days	phase 1/2	*est. enrollment: 60<br>*nabpaclitaxel (100-250 mg/m2) and cyclophosphamide (15-35 mg/kg)	*Feng et al. 2016, only NSCLC: 2/11PR, 5/11SD, 4/11PD<br>*Guo et al. 2018, only advanced biliary tract cancers (14 cholangiocarcinomas and 5 gallbladder carcinomas):<br>*1/17 CR, 10/17 SD<br>*The median progression-free survival was 4 months.	NCT01869166; status unknown;<br>PI: Weidong Han	[37-39]	Chinese PLA General Hospital, China	*Feng et al., 2016: Mild skin toxicity, nausea, vomiting, dyspnea, andhypotension; one patient suffered from a transient grade 3/4 increase in serum lipase.<br>*On-target/off-tumor toxicity: serum lipase increase<br>*Feng et al., 2017: EGFR: mild chills, fever, fatigue, vomiting and muscle soreness, and a 9-day duration of delayed lower fever, accompanied by escalation of IL-6 and CRP, acute increase of glutamic-pyruvic transaminase and glutamic-oxalacetic transaminase, and grade 2 lichenstriatus-like skin pathological changes.<br>*Guo et al., 2018:<br>*infusion was tolerated;grade≥3 lymphopenia (10 of 19); grade≥3 thrombocytopenia (2 of 19); 3 patients grade>=3 acute fever/chill<br>*grade 1/2 target-mediated toxicities (mucosal/cutaneous toxicities and acute pulmonary edema (increase of IL-6 and CRP))
EGFR	NSCLC	*CXCR5-modified<br>*Autologous T cells	?	iv	The first dose group: 0.5×10^6/kg CAR positive T cells; the second dose group: 1.58×10^6/kg CAR positive T cells; the third dose group: 5×10^6/kg CAR positive T cells. For subjects with body weight greater than 60 kg, the number of cells can only be calculated according to 60 kg of body weight.	phase 1	est. enrollment: 11		NCT04153799; recruiting;<br>PI: Li Zhang	[40]	*Sun Yat-sen UniversityGuangzhou<br>*Bio-gene Technology Co., Ltd., China	
EGFR	EGFR-family-member-positive, advanced solid tumor:<br>*lung,<br>*liver,<br>*stomach	*HerinCAR-PD1<br>*PD-1 antibody expressing CAR-T	?	iv	3 cycles<br>$1\text{-}5x10^7$/kg	phase 1/2	*est. enrollment: 20		NCT02862028; unknown;<br>PI: Naiyan Han		shanghai international medical center, China	
EGFR	Metastatic colorectal cancer	*4th generation (TRUCK)<br>*scFv-4-1BB/CD28/CD3ζ<br>*IL-12 expression under control of NFAT	?	?	MTD determination	phase 1/2	est. enrollment: 20		NCT03542799; not yet recruiting;<br>PI: Geng Tian		Shenzhen second people's hospital, China	
EGFR	Malignant gliomas	?	lentiviral	iv	?	phase 1	*est. enrollment: 10<br>*Aldesleukin<br>*non-myeloablative conditioning (cy+flu)		NCT02331693; unknown;<br>PI: not provided		RenJi Hospital, China	
EGFR	Metastatic colorectal cancer	scFv-4-1BB/CD28/CD3ζ	?	?	MTD determination	phase 1/2	est. enrollment: 20		NCT03152435; unknown;<br>PI: Geng Tian		Shenzhen second people's hospital, China	
EGFRvIII	Malignant glioma	scFv-CD28/4-1BB/CD3ζ	retroviral	iv	Phase 1: dose escalation until MTD (maximum tolerated dose) determined, then phase 2	phase 1/2	*est. enrollment: 107<br>*actual enrollment: 18<br>*non-myoablative, but lymphodepleting conditioning regimen(cy+flu)<br>*with aldesleukin (IL-2; also iv, 72,000IU/kg)<br>*many events	*Combined steroids/ no steroids, Cohort 8: participant experienced a treatment-related mortality (TRM).	NCT01454596; completed;<br>PI: SA Rosenberg	[45,229,230]	NCI, NIH, USA	On-target/off-tumor toxicity:<br>ARDS (one treatment-related death)
EGFRvIII	Glioblastoma	*scFv-CD8hinge/tm-4-1BB/CD3ζ<br>*scFv based on 3C10, humanized	lentiviral	iv	$1\text{-}5x10^8$	phase 1	*est. enrollment: 12<br>*Temozolomide, radiation	*Manufacturing and infusion are feasible and safe<br>*no off-tumor toxicity, no CRS<br>*O'Rourke et al.: 2/10PD, 1/10SD, 7/10 died	NCT02209376; Terminated (Sponsor decision to terminate prior to completion to pursue combination therapies. NCT03726515);<br>PI: Donald O'Rourke, Susan Chang	[231-234]	*Upenn<br>*University of California, San Francisco, USA	*Seizure, headache, weakness,cerebral edema<br>*On-target/off-tumor toxicity: no
EGFRvIII	Recurrent glioblastoma multiforme	?	lentiviral	iv	*3+3 dose escalation<br>*$5x10^4$/kg-$1x10^7$/kg	phase 1	*est. enrollment: 20<br>*cy+flu		NCT02844062; unknown;<br>PI: Zhixiong Lin		Sanbo Brain Hospital capital medical university, China	
EGFR family member	Advanced solid tumors	*herinCAR-PD1 cells<br>*PD-1 antibody expressing	?	iv	$1\text{-}5x10^7$/kg herin CAR-PD1 + physiological saline + 0.25% human alloalbumin (300ml)	phase 1/2	*est. enrollment: 20		NCT02873390; unknown;<br>PI: Jiangtao Wang		Ningo No.5 Hospital, China	
EGFRvIII	Newly-diagnosed WHO grade IV malignant glioma	?	retroviral	iv	single infusion, MTD<br>$4.5x10^6$/kg<br>$1.5x10^7$/kg<br>$4.5x10^7$/kg<br>$1.5x10^8$/kg	phase 1	*actual enrollment: 3<br>*lymphodepletion with temozolomide		NCT02664363; Terminated (Study funding ended);<br>PI: David Ashley, Daniel Landi	[203]	Duke University Hospital, USA	
EGFR	EGFR-positive advanced solid tumors	CTLA-4 and PD-1 antibodies expressing	?	iv	$2\text{-}5x10^7$ cells/kg	phase 1/2	*est. enrollment: 40<br>*cyclophosphamide		NCT03182816; unknown;<br>PI: Jiangtao Wang		Ningbo No.5 Hospital, China	

Antigen	Cancer	CAR type<br>/add-ons	CAR transfer	Mode of injection	Number of cells	Phase	Patient #<br>/pretreatment<br>/other drugs	Outcome	NCT#<br>/Status<br>/PI	Ref	Origin	Adverse events
EGFR	Recurrent/refractory solid tumors in children and young adults:<br>*germ cell tumor,<br>*retinoblastoma,<br>*hepatoblastoma,<br>*wilms tumor,<br>*rhabdoid tumor,<br>*carcinoma,<br>*osteosarcoma,<br>*ewing sarcoma,<br>*rhabdomyosarcoma,<br>*synovial sarcoma,<br>*clear cell sarcoma,<br>*malignant peripheral nerve sheath tumors,<br>*desmoplastic small round cell tumor,<br>*soft tissue sarcoma,<br>*neuroblastoma	*scFv-4-1BB/CD3ζ<br>*In second arm also CD19 CAR-T (there with HER2tG selection marker)<br>*Selection suicide marker EGFRt	lentiviral	?	?	phase 1	est. enrollment: 36		NCT03618381;<br>recruiting;<br>PI: Katie Albert	[41]	Seattle Children's Hospital, USA	
EGFRvIII	Glioblastoma multiforme	*4SCAR-IgT cells<br>*Secreting PD1 and PD-L1 antibodies	?	iv or it	*3+3 dose escalation<br>*1x10^5/kg-1x10^7/kg	phase 1	*est. enrollment: 20<br>*cy+flu		NCT03170141;<br>enrolling by invitation;<br>PI: Lung-Ji Chang		Shenzhen Geno-Immune Medical Institute, China	
EGFRvIII	GBM	*scFv-4-1BB/CD3ζ<br>*scFv based on 3C10, humanized	?	?	?	phase 1	*est. enrollment: 7<br>*Pembro		NCT03726515;<br>Active, not recruiting;<br>PI: Donald O'Rourke		Abramson Cancer Center of the Upenn, USA	
EGFRvIII	Recurrent GBM	?	retroviral	intracerebral (it)	*2.5x10^8 starting dose,<br>*dose escalation	phase 1	est. enrollment: 24		NCT03283631;<br>Suspended (We are temporally halting enrollment until a protocol amendment is approved.);<br>PI: Daniel Landi, David Ashley		Duke University Hospital, USA	
EGFR806	EGFR-positive recurrent or refractory pediatric CNS tumors	* also expressing EGFRt	lentiviral	Tumor resection cavity or ventricular system	*dose escalation	phase 1	*est. enrollment: 36		NCT03638167;<br>recruiting;<br>PI: Gust		Seatle Children's Hospital, USA	
EpCAM	EpCAM-positive cancer:<br>*colon,<br>*esophageal,<br>*pancreatic,<br>*prostate,<br>*gastric,<br>*hepatic	scFv-CD28/CD3ζ	lentiviral	Vascular interventional mediated or endoscopy	1-10x10^6 EpCAM-CAR positive T cells/kg	phase 1/2	*est. enrollment: 60<br>*preconditioning		NCT03013712;<br>recruiting;<br>PI: Xiao-an Li		First affiliated hospital of Chengdu Medical College, China	
EpCAM	Liver cancer	?	?	?	5 doses	phase 2	*est. enrollment: 25		NCT02729493;<br>unknown;<br>PI: Xianping Cheng		Sinobioway cell therapy Co. Ltd., China	
EpCAM	Stomach cancer	?	?	?	5 doses	phase 2	*est. enrollment: 19		NCT02725125;<br>Unknown;<br>PI: Yifu He		Sinobioway cell therapy Co. Ltd., China	
EpCAM	Advanced gastric cancer with peritoneal metastasis	?	?	ip	?	phase 1	*est. enrollment: 40		NCT03563326;<br>recruiting;<br>PI: Jian-Kun Hu		West China Hospital, Sichuan University, China	
EpCAM	*Nasopharyngeal carcinoma<br>*breast cancer	3rd generation	lentiviral	?	dose escalation until MTD	phase 1	*est. enrollment: 30<br>*cyclophosphamide		NCT02915445;<br>recruiting;<br>PI: Wie Wang		West China Hospital, Sichuan University, China	
EphA2	Malignant glioma	?	?	?	?	phase 1/2	*actual enrollment: 60<br>*no preconditioning		NCT02575261;<br>completed;<br>PI: Lizhi Niu		Fuda Cancer Hospital, Guangzhou, China	
ErbB dimers (HER2, 3 and EGFR)	SCCHN (squamous cell cancer of the head and neck)	*T1E-CD28/CD3ζ: CAR coupling a promiscuous ErbB ligand derived from EGF and TGFα to a fused CD28+CD3ζ endodomain<br>*4αβ, a chimeric cytokine receptor containing the IL-4Rα ectodomain coupled to the IL-2Rβ endodomain	retroviral	it	*dose escalation<br>*10^7-10^9 transduced T4+ T cells	phase 1	*est. enrollment: 30<br>*lower dose without lymphodepletion, higher dose with	CAR-T Congress EU 2019: 9/15 SD, 1/15 CR	NCT01818323;<br>recruiting,;<br>PI: John Maher	[142-144,190]	King's College London, UK	
FAP	Malignant pleural mesothelioma	*scFv-CD28/CD3ζ<br>*scFv based on humanized form of F19 (sibrotuzumab)	retroviral	ip	1x10^6 single dose	phase 1	*actual enrollment: 4<br>*lymphodepletion		NCT01722149,;<br>completed,,<br>PI: Alessandra Curioni	[131,132]	University Hospital Zurich, Switzerland	
FBP (FRα; autologous folate receptor-alpha)	Recurrent high grade serous ovarian cancer	MOv19-4-1BB/CD3ζ	lentiviral	ip	*Cohort 1: single infusion 1-3x10^7/m^2 without lympodep.<br>*Cohort 2: single infusion 1-3x10^7/m^2 with lympodep.<br>*Cohort 3: single infusion 1-3x10^8/m^2 with lympodep.<br>*Cohort -1: single infusion 1-3x10^6/m^2 without lympodep.	phase 1	*est. enrollment: 18<br>*w/ or w/o cy+flu		NCT03585764;<br>recruiting;<br>PI: Payal D Shah		University of Pennsylvania, Philadelphia, USA	
FBP (folate binding protein) (FRα)	Ovarian epithelial cancer	*MOv-gamma-PBL<br>*scFv-FcεRIγ<br>*scFv based on MOv18	retroviral	iv	*Dose escalation until MTD<br>*0.3-5x10^{10}	phase 1	*with/without IL-2 added<br>*no preconditioning	No reduction in tumor burdenwas seen in any patient (n=14)	NCT00019136;<br>completed,,<br>PI: Steven Rosenberg	[235]	NCI, USA	*One patient reported no severe CAR-T cell related toxicities. 12 patients experienced grade 1 or 2 toxicity events and 5 patients experienced grade3–4 toxicity events.<br>*Grade 3/4 toxicities including hypotension and dyspneaas well as less frequently fatigue, leukopenia, rigors, sinustachycardia, and diarrhea, in some patients receiving IL-2<br>*On-target/off-tumor toxicity: no

Antigen	Cancer	CAR type /add-ons	CAR transfer	Mode of injection	Number of cells	Phase	Patient # /pretreatment /other drugs	Outcome	NCT# /Status /PI	Ref	Origin	Adverse events
GD2	Brain tumors (high grade glioma (HGG) or diffuse intrinsic pontine glioma (DIPG))	*Constitutively active IL-7R	retroviral	iv	*DL0: 1x10^{7} GD2 CAR-T (without C7R)<br>*DL1: 1x10^{7} C7R-GD2 CAR-T<br>*DL2: 3x10^{7} C7R-GD2 CAR-T<br>*DL3: 1x10^{8} C7R-GD2 CAR-T	phase 1	*est. enrollment: 34<br>*cy+flu		NCT04099797; recruiting;<br>PI: Bilal Omer, MD	[188]	Baylor College of Medicine, USA	
GD2	*Relapsed/refractory neuroblastoma<br>*Uveal melanoma	*C7R-GD2.CAR-T<br>*constitutively active IL-7R	retroviral	iv	*Dose level 0: 1x10^{7} CAR-T w/o lymphodep.<br>*Dose level 1: 1x10^{7} CAR-T w/ lymphodep.<br>*Dose level 2: 3x10^{7} CAR-T w/ lymphodep.<br>*Dose level 3: 1x10^{8} CAR-T w/ lymphodep.	phase 1	*est. enrollment: 34<br>*lymphodepletion: cy+flu		NCT03635632; recruiting;<br>PI: Bilal Omer	[188]	Baylor College of Medicine, USA	
GD2	*Glioma of spinal cord<br>*Glioma of brainstem	*scFv-4-1BB/CD3ζ<br>*iCasp9	retroviral	infusion?	*3+3 dose escalation, subjects with H3K27M-mutant DIPG or spinal DMG, starting with Dose Level 1<br>*Dose Level 1: 1x10^{6} CAR+ T cells/kg<br>*Dose Level 2: 3x10^{6} CAR+ T cells/kg<br>*Dose Level 3: 10x10^{6} CAR+ T cells/kg	phase 1	*est. enrollment: 54<br>*cy+flu		NCT04196413; recruiting;<br>PI: Michelle Monje		Lucile Packard Children's Hospital (LPCH), Stanford, USA	
GD2	*GD2+ solid tumors:<br>*sarcoma,<br>*neuroblastoma,<br>*melanoma<br>*osteosarcoma	*scFv-OX40/CD28/CD3ζ<br>*ICD9<br>*scFv based on 14g2a	retroviral	iv	escalating doses<br>*dose 1: 1x10^{5} cells/kg<br>*dose 2: 1x10^{6} cells/kg<br>*dose 3: 3x10^{6} cells/kg<br>*dose 4: 1x10^{7} cells/kg	phase 1	*original est. enrollment: 72<br>*actual enrollment: 15<br>*cy+flu	In Gargett et al., : formelanoma 4/4 PR	NCT02107963; completed;<br>PI: Rosandra Kaplan	[235-238]	NCI, NIH, USA	On-target/off-tumor toxicity: no
GD2	Relapsed or refractory neuroblastoma in children	?	?	?	?	phase 1/2	*est. enrollment: 22		NCT02919046; recruiting;<br>PI: Yongjun Fang, Kuiran Dong		Nanjing Children's Hospital, China	
GD2	Refractory or metastatic GD2-positive:<br>*sarcoma<br>*neuroblastoma	*iC9-GD2-CAR-VZV-CTLs (in combination with VZV vaccination) (transgenic VZV-specific T cells)<br>*scFv-OX40/CD28/CD3ζ<br>*iCasp9<br>*scFv based on 14g2a	retroviral	iv	DL1: 1x10^{6} cells/m^{2}<br>DL2: 1x10^{7} cells/m^{2}<br>DL7: first VZV vaccine then 1x10^{7} cells/m^{2}<br>DL8: first VZV vaccine then 1x10^{8} cells/m^{2}<br>DL9: first lymphodepletion then 1x10^{8} cells/m^{2}<br>DL10: first lymphodepletion then 5x10^{8} cells/m^{2}<br>DL11: first lymphodepletion then 1x10^{9} cells/m^{2}	phase 1	*est. enrollment: 26<br>*lymphodepletion: cy+flu		NCT01953900; Active, not recruiting;<br>PI: Lisa Wang, Sarah Whittle, Cliona Rooney	[41,159]	Baylor College of Medicine, USA	
GD2	Recurrent neuroblastoma	4th generation	lentiviral	?	>=2x10^{5} cells/kg	phase 2	*est. enrollment: 30<br>*cy+flu		NCT02765243; recruiting;<br>PI: Lihua Yang		Southern Medical University, China	
GD2	GD2+ solid tumors	*4th generation<br>*inducible apoptotic caspase 9 domain	lentiviral	?	?	phase 1/2	*est. enrollment: 100<br>*cy+flu		NCT02992210; recruiting;<br>PI: Lung-Ji Chang		Shenzhen Geno-Immune Medical Institute, China	
GD2	Relapsed/refractory melanoma	*scFv-CD3ζ<br>*vaccine-specific T cells<br>*scFv based on 14g2a	retroviral	?	?	phase 1	est. enrollment: 3		NCT02482532; recruiting;<br>PI: Gary Doolittle		KU Cancer Center, Fairway, Kansas, USA	
GD2	Neuroblastoma	*scFv-CD3ζ<br>*In EBV-specific T cells<br>*scFv based on 14g2a	retroviral	iv	2x10^{7} cells/m^{2}<br>5x10^{7} cells/m^{2}<br>1x10^{8} cells/m^{2}<br>single infusion	phase 1	*actual enrollment: 19<br>*without lymphodepletion!	3/19 CR, 1/19 PR, 8/19 NED (no evidence of disease), 6/19PD, 1/19SD, 2/19 tumor necrosis, 1/19 relapse	NCT00085930; active, not recruiting;<br>PI: Andras A Heczey	[160,161]	*Texas Children's Hospital, Houston, Texas<br>*Baylor College of Medicine, USA	*Mild to moderate local pain at the site of tumor, necrosis in two patients<br>*On-target/off-tumor toxicity: no
GD2	Relapsed/refractory neuroblastoma	*3rd generation<br>*Expressing IL-15<br>*iCasp9 safety switch	?	?	dose escalation 0,5x10^{6}, 1x10^{6}, 1.5x10^{6} cells/kg	phase 1	*est. enrollment: 18<br>*cy+flu		NCT03721068; recruiting;<br>PI: George Hucks		UNC Lineberger Comprehensive Cancer Center, USA	
GD2	Relapsed and refractory neuroblastoma	scFv-CD28/CD3ζ	?	iv	DL1: 1x10^{7} CAR-T/m^{2}<br>DL2: cyclophosphomide and 1x10^{7} CAR-T/m^{2}<br>DL3: cy+flu and 1x10^{7} CAR-T/m^{2}<br>DL4: cy+flu and 1x10^{8} CAR-T/m^{2}<br>DL5: cy+flu and 5-10x10^{8} CAR-T/m^{2}	phase 1	*est. enrollment: 27<br>*cy+flu	No clinical responses in first 12 patients, but response in many sites of bone marrow disease for 1 patient.	NCT02761915; recruiting;<br>PI: not provided	[239]	Cancer Research UK	
GD2	Pediatric relapsed/refractory neuroblastoma	*iCasp9	?	?	1-10x10^{6}/kg CAR+ T cells, single dose	phase 1/2	*est. enrollment: 42<br>*lymphodepletion		NCT03373097; recruiting;<br>PI: unknown		Bambino Gesu Hospital and Research Institute, Italy	
GD2	GD2+ glioma	?	?	infusion?	?	phase 1/2	*actual enrollment: 60		NCT03252171; completed;<br>PI: Lizhi Niu		Fuda Cancer Hospital, China	
GD2	Relapsed/refractory neuroblastoma	*scFv-CD3ζ<br>*In allogeneic, multivirus-specific CTL after allogeneic HSCT<br>*scFv based on 14g2a	retroviral	?	A single infusion of 2x10^{6}/m^{2}	phase 1	*actual enrollment: 5<br>*submyeloablative conditioning regimen	*no toxicity vs. patient<br>*3 evaluable: non-complete response: 3	NCT01460901; completed;<br>PI: Doug Myers		Children's Mercy Hospital Kansas City, USA	
GD2	Neuroblastoma	*3rd generation (CD28 and OX40)<br>*with iCaspase suicide safety switch	retroviral	iv	1x10^{7}, 1x10^{8}, 2x10^{8} cells/m^{2}	phase 1	*actual enrollment: 11<br>* w/ and w/o cy+flu<br>*w/ and w/o Pembro	3/11 SD, 8/11 PD	NCT01822652; active, not recruiting;<br>PI: Andras A. Heczey	[199]	Baylor College of Medicine, USA	Grade 3 or higher: fever and neutropenia, anemia, leukopenia, lymphopenia, neutropenia, thrombocytopenia, extremity pain

Antigen	Cancer	CAR type /add-ons	CAR transfer	Mode of injection	Number of cells	Phase	Patient # /pretreatment /other drugs	Outcome	NCT# /Status /PI	Ref	Origin	Adverse events
GD2	Glioma	?	?	iv		phase 1/2	actual enrollment: 2		NCT04406610;<br>terminated;<br>PI: ?		Fuda Cancer Hospital, Guangzhou, China	
gp100(209-217) /HLA-A2	Malignant melanoma	*TCR-like antibody-CAR-4-1BB/CD3ζ<br>*GPA-TriMAR (binding gp100/HLA-A2)<br>*extracellular 3 subunits, one binds antigen, other two function to stimulate the innate immune system	lentiviral	?	?	early phase 1	*est. enrollment: 6		NCT03649529,;<br>recruiting;<br>PI: Haifeng Lin	[155,156]	Second affiliated hospital of Hainan Medical University, China	
GPC3 (glypican-3)	HCC	?	?	?	every two weeks<br>DL1: $1x10^7/m^2$<br>DL2: $3x10^7/m^2$<br>DL3: $1x10^8/m^2$	phase 1	*est. enrollment: 20		NCT04121273;<br>recruiting;<br>PI: not provided		The Affiliated Nanjing Drum Tower Hospital of Nanjing University Medical School, China	
GPC3 (glypican-3)	Liver cancer	*2nd generation<br>*Co-expressing cytokines IL-21 and IL-15<br>*scFv based on GC33	retroviral	iv	MTDDL1: $1x10^7/m^2$DL2: $3x10^7/m^2$DL3: $1x10^8/m^2$DL4: $3x10^8/m^2$DL5: $1x10^9/m^2$	phase 1	*est. enrollment: 60<br>*cy+flu		NCT04093648;<br>Withdrawn (The key elements of this study were incorporated into another study.);<br>PI: Andras Heczey, MD		Baylor College of Medicine, USA	
GPC3 (glypican-3)	Advanced HCC	4th generation	?	iv	*MTD<br>*3 dose levels<br>*3+3 dose escalation<br>*at dose level 3:<br>Cohort 1: combined with tyrosine kinase inhib.<br>Cohort 2: combined with PD-1/PD-L1 antibodies	phase 1	*est. enrollment: 36<br>*cy+flu		NCT03980288;<br>recruiting;<br>PI: Tingbo Liang		The First Affiliated Hospital, Zhejiang University, China	
GPC3 (glypican-3)	Advanced HCC	?	?	iv	MTD; the subjects are enrolled into 2 dose levels cohorts in sequence	phase 1	*est. enrollment: 15<br>*lymphodepleting pretreatment		NCT03884751;<br>recruiting;<br>PI: Qin shukui; Zhai bo		The 81st Hospital of PLA<br>RenJi Hospital, China	
GPC3 (glypican-3)	Recurrent or refractory lung squamous cell carcinoma	scFv-CD28/CD3ζ	lentiviral	iv	dose escalation: $1x10^5$-$2x10^9$ CAR-GPC3 T cells/kg	phase 1	*est. enrollment: 20<br>*cy+flu		NCT02876978;<br>unknown;<br>PI: Jiang Liyan		Shanghai Chest Hospital, Shanghai Jiaotong University, China	
GPC3 (glypican-3)	Refractory HCC	scFv-CD28/CD3ζ	lentiviral	iv	$20x10^8$ single dose	phase 1	*est. enrollment: 20<br>*cy+flu	3/5 PD, 1/5 NE, 1/5 PR	NCT03146234;<br>completed;<br>PI: Bo Zhai	[25,240]	RenJi Hospital, China	Decrease WBC, platelet count, lymphocyte count, neutrophil count, Pyrexia, increased APTT, AST, and ALT, bilirubin level increased, renal impairment, hypotension
GPC3 (glypican-3)	GPC3+ cancer:<br>*HCC,<br>*squamous cell lung cancer	*3rd generation<br>*GPC3-T2-CAR (GPC3 and/or GPC3 and soluble TGFbeta targeting)<br>*IL-7/CCL19 producing	?	iv, it	?	phase 1	est. enrollment: 30		NCT03198546;<br>recruiting;<br>PI: Zhengfeng Zhang	[183]	Second affiliated hospital of Guangzhou medical university, China	
GPC3 (glypican-3)	Advanced HCC	scFv-4-1BB/CD3ζ	transduction ?	it	1-$10x10^6$ CAR+ T cells	phase 1/2	*est. enrollment: 10		NCT03130712;<br>unknown;<br>PI: Lu Yinying		Beijing 302 Hospital, China	
GPC3 (glypican-3)	GPC3+ advanced HCC	*scFv-4-1BB/CD3ζ<br>*tEGFR	retroviral	iv, TAI	?	phase 1/2	*est. enrollment: 20<br>*transcatheter arterial chemoembolization (TACE)<br>*cy+flu		NCT03084380;<br>not yet recruiting;<br>PI: Qingzhu Jia		Xinqiao Hospital of Chongqing, China	
GPC3 (glypican-3)	HCC	scFv-CD28/4-1BB/CD3ζ	retroviral	?	dose escalation:<br>DL1: $1x10^7/m^2$<br>DL2: $3x10^7/m^2$<br>DL3: $1x10^8/m^2$<br>DL4: $3x10^8/m^2$<br>DL5: $1x10^9/m^2$	phase 1	*est. enrollment: 14<br>*cy+flu		NCT02905188;<br>recruiting;<br>PI: Andras Heczey	[134]	Baylor College of Medicine, USA	
GPC3 (glypican-3)	HCC	?	?	?	?	phase 1/2	actual enrollment: 60		NCT02723942;<br>completed;<br>PI: Lizhi Niu		Fuda Cancer Hospital; Guangzhou, China	
GPC3 (glypican-3)	Pediatric solid tumors (GAP)	scFv based on GC33	retroviral	?	dose escalation:<br>DL1: 1x107/m2<br>DL2: 3x107/m2<br>DL3: 1x108/m2<br>DL4: 3x108/m2<br>DL5: 1x109/m2	phase 1	*est. enrollment: 18<br>*cy+flu		NCT02932956;<br>recruiting;<br>PI: Heczey		Baylor College of Medicine, USA	
GPC3 (glypican-3)	Advanced HCC	scFv-4-1BB/CD3ζ	?	TAI	1-$10x10^6$ CAR+<br>T cells/kg	Phase 1/2	*est. enrollment: 30<br>*single dose of cyclophosphamide ($1.5grams/m^2$)		NCT02715362;<br>unknown;<br>PI: Xu Aimin		RenJi Hospital, China	
GPC3 (glypican-3)	*Pediatric solid tumors<br>*Liver cancer<br>*Rhabdomyosarcoma<br>*Malignant rhabdoid tumor<br>*Liposarcoma (soft tissue)<br>*Wilms tumor<br>*Yolk sac tumor (germ cell tumor)	*AGAR T cells<br>*IL-15 armored<br>*iCasp9	retroviral	iv	DL1: $3x10^7/m^2$<br>DL2: $1x10^8/m^2$<br>DL3: $3x10^8/m^2$<br>DL4: $1x10^9/m^2$	phase 1	*est. enrollment: 24<br>*cy+flu		NCT04377932;<br>not yet recruiting;<br>PI: Andras Heczey and David Steffin		Baylor College of Medicine, USA	
GPC3 (glypican-3)	Advanced HCC	scFv-CD28/CD3ζ	lentiviral	iv	$7x10^8$-$92.5x10^8$, split dose	phase 1	*actual enrollment: 13<br>*either cy only, or cy+flu	2/8 SD, 1/8 PR, 3/8 NE, 2/8 PD	NCT02395250;<br>completed;<br>PI: Bo Zhai	[25,240]	RenJi Hospital, China	Decrease WBC, platelet count, lymphocyte count,pyrexia, increased APTT, AST, and ALT, bilirubin level increased
GPC3 (glypican-3)	Previously treated solid tumors	TAK-102	?	iv	Cohort 1: 1×10^7 CAR (+) cells/body [starting dose].<br>Cohort 2: 1×10^8 CAR (+) cells/body.<br>Cohort 3: 1×10^9 CAR (+) cells/body.	phase 1	*est. enrollment: 18		NCT04405778;<br>not yet recruiting;<br>PI: ?		Takeda, Japan	

Antigen	Cancer	CAR type /add-ons	CAR transfer	Mode of injection	Number of cells	Phase	Patient # /pretreatment /other drugs	Outcome	NCT# /Status /PI	Ref	Origin	Adverse events
HER2	*Chemotherapy refractory HER-2+ advanced solid tumors:<br>*biliary tract cancers,<br>*pancreatic cancers	*scFv-CD3ζ<br>*scFv-4-1BB/CD3ζ	lentiviral	iv	1 to 2 cycles of CAR-T-HER2 (median CAR+ T cells $2.1x10^6$/kg); dose escalation over several days	phase 1/2	*actual enrollment: 11<br>*nabpaclitaxel (100-200 mg/m2) and cyclophosphamide (15-35 mg/kg)	*1/11 PR, 5/11 SD, 5/11 PD	NCT01935843;<br>status unknown;<br>PI: Weidong Han	[44]	Chinese PLA General Hospital, China	*Mild-to-moderate fatigue, nausea, vomiting, myalgia, arthralgia, and lymphopenia,<br>*Except 1 grade-3 acute febrile syndrome and 1 abnormal elevation of transaminase<br>*post-infusion toxicities: 1 case of reversible severe upper gastrointestinal hemorrhage (metastasis), and 2 cases of grade 1-2 delayed fever (release of C-reactive protein and IL-6)<br>*On-target/off-tumor toxicity: Liver enzymes increase
HER2	*Bladder cancer<br>*Head and neck squamous cell carcinoma<br>*Cancer of the salivary gland<br>*Lung cancer<br>*Breast cancer<br>*Gastric cancer<br>*Esophageal cancer<br>*Colorectal cancer<br>*Pancreatic adenocarcinoma	*HER2 CAR-modified adenovirus-specific CTL<br>*with oncolytic adenovirus injected intratumorally	?	?	*Dose Level 1<br>CAdVEC = $1x10^{10}$<br>HER2.CAR-AdVST = 0<br>*Dose Level 2<br>CAdVEC = $1x10^{10}$<br>HER2.CAR-AdVST = $1x10^6$<br>*Dose Level 3<br>CAdVEC = $1x10^{11}$<br>HER2.CAR-AdVST = $1x10^6$<br>*Dose Level 4<br>CAdVEC = $1x10^{11}$<br>HER2.CAR-AdVST = $1x10^7$<br>*Dose Level 5<br>CAdVEC = $1x10^{12}$<br>HER2.CAR-AdVST = $1x10^7$<br>*Dose Level 6<br>CAdVEC = $1x10^{12}$<br>HER2.CAR-AdVST = $1x10^8$	phase 1	est. enrollment: 39		NCT03740256;<br>not yet recruiting;<br>PI: Andrew Sikora		Texas Children's Hospital, USA	
HER2	Recurrent/refractory grade III-IV glioma	*scFv-4-1BB/CD3ζ<br>*CD19t<br>*in memory-enriched T cells	lentiviral	ic, it, or intraventricular, or both	?	phase 1	est. enrollment: 51		NCT03389230;<br>recruiting;<br>PI: Behnam Badie		City of Hope Medical Center, USA	
HER2	Colon cancer	*scFv-CD28/4-1BB/CD3ζ<br>*scFv based on Herceptin (Trastuzumab; humanized)	retroviral	iv	$1x10^{10}$	phase I/II	*actual enrollment: 1<br>*lymphodepletion (cy+flu)<br>*Aldesleukin (IL-2)	1 treated, 1 dead CRS due to low expression of ERBB2 (HER2) on lung	NCT00924287,;<br>Terminated (This study was terminated after the first patient treated on study died as a result of the treatment);<br>PI: Steven A Rosenberg	[12]	NCI, NIH, USA	*Respiratory distress and dramatic pulmonary infiltrate on chest X-ray were observed soon after CAR-T cell administration.<br>*Severe hypotension, bradycardia, gastrointestinal bleeding, resulting in a cardiac arrest.<br>*The patient died 5 days after the CAR-T infusion.<br>*On-target/off-tumor toxicity: ARDS (fatal)
HER2	HER2-positive recurrent/refractory pediatric CNS tumors	*Third generation<br>*EGFRt	lentiviral	tumor resection cavity or ventricular system	?	phase 1	*est. enrollment: 36		NCT03500991;<br>recruiting;<br>PI: Nicholas Vitanza		Seattle Children's Hospital, USA	
HER2	Her2+ malignancy	*scFv-CD28/CD3ζ<br>*TGFβ dominant negative receptor (TGFβ resistant)<br>*EBV-specific through native TCR	retroviral	iv	single dose<br>level 1: $1x10^4$ cells/m^2<br>level 2: $3x10^4$ cells/m^2<br>level 5: $1x10^6$ cells/m^2<br>level 6: $3x10^6$ cells/m^2<br>level 7: $1x10^7$ cells/m^2<br>level 8: $3x10^7$ cells/m^2<br>level 9: $1x10^8$ cells/m^2	phase 1	*actual enrollment: 20<br>*no preconditioning		NCT00889954;<br>completed;<br>PI: Stephen Gottschalk		*Baylor College of Medicine<br>*Texas Children's Hospital, USA	
HER2	Glioblastoma	2nd generation	retroviral	it or tumor resection cavity	dose escalation<br>-$1x10^7$<br>-$3x10^7$<br>-$1x10^8$	phase 1	*est. enrollment: 14		NCT02442297;<br>recruiting;<br>PI: Nabil Ahmed		*Baylor College of Medicine<br>*Texas Children's Hospital, USA	
HER2	HER2+ brain or leptomeningeal metastases	*scFv-4-1BB/CD3ζ<br>*memory-enriched<br>*CD19t	?	intraventricular	3 doses	phase 1	est. enrollment: 30		NCT03696030;<br>recruiting;<br>PI: Jana Portnow		City of Hope Medical Center, USA	
HER2	HER2+ glioblastoma multiforme	*scFv-CD28/CD3ζ<br>*in CMV-specific T cells selected	retroviral	iv	*one dose:-$1x10^6/m^2$, or-$3x10^6/m^2$, or -$1x10^7/m^2$, or-$3x10^7/m^2$, or-$1x10^8/m^2$<br>*if beneficial, patient can get up to 6 additional same doses	phase 1	*actual enrollment: 16<br>*no preconditioning	1/16 PR, 7/16 SD, 8/16 PD	NCT01109095;<br>Completed;<br>PI: Ahmed	[45-47]	*Baylor College of Medicine<br>*Texas Children's Hospital, USA	*No severe adverse effects or cytokine release syndrome within the used doses<br>*On-target/off-tumor toxicity: no
HER2	Breast cancer	scFv-CD28/CD3ζ	retroviral	?	?	phase I/II	*actual enrollment: 60<br>*preconditioning		NCT02547961;<br>completed;<br>PI: Lizhi Niu		Fuda Cancer Hospital, Guangzhou, China	
HER2	*Breast,<br>*ovarian,<br>*lung,<br>*gastric,<br>*colorectal,<br>*glioma,<br>*pancreatic	?	?	?	determine MTD	phase 1/2	est. enrollment: 60		NCT02713984;<br>Withdrawn (Reform CAR structure due to safety consideration);<br>PI: Cheng Qian		Southwest Hospital, China	
HER2	Advanced sarcoma	*scFv-CD28/CD3ζ<br>*scFv based on FRP5	retroviral	iv	DLTDose levels of $1x10^4/m^2$ to $1x10^8/m^2$ with or without lymphodepletion	phase 1	*est. enrollment: 36<br>*lymphodepletion: fludarabine with or without cyclophosphamide	*without preconditioning: 4/17 SD, 12/17 PD, 1/17 PR<br>*with preconditioning: 2/10 CR, 3/10 SD	NCT00902044;<br>Active, not recruiting;<br>PI: Nabil Ahmed	[48-50]	Baylor College of Medicine, USA	*well tolerated, no systemic side effects<br>*Fever observed in 1 patient*On-target/off-tumor toxicity: no
ICAM1	*Anaplastic thyroid cancer*Thyroid cancer	*AIC100<br>*no scFv, but I-domain of CD11a of LFA-1, linked to CD8TM-CD28/4-1BB/CD3ζ	transfection?	iv	*Cohort -1 AIC100 Cell Dose Level -1 (Flat Dose): 1 x 10^6 cells<br>*Cohort 1 AIC100 Cell Dose Level 1 (Flat Dose): 1 x 10^7 cells<br>*Cohort 2 AIC100 Cell Dose Level 2 (Flat Dose): 1 x 10^8 cells<br>*Cohort 3 AIC100 Cell Dose Level 3 (Flat Dose): 5 x 10^8 cells	phase 1	*est. enrollment: 24<br>*lymphodepleting therapy		NCT04420754;<br>not yet recruiting;<br>PI: Koen van Besien		Weill Medical College of Cornell University, USA	

Antigen	Cancer	CAR type<br>/add-ons	CAR transfer	Mode of injection	Number of cells	Phase	Patient #<br>/pretreatment<br>/other drugs	Outcome	NCT#<br>/Status<br>/PI	Ref	Origin	Adverse events
IL13Rα2	r/r glioblastoma	*IL13 (E13Y)-hinge-optimized-4-1BB/CD3ζ (no scFv, but binding-optimized IL-13)<br>*truncated CD19	?	icr, intraventricular /icr, it	?	phase 1	*est. enrollment: 60<br>*with and without nivo and ipi before or after CAR-T application		NCT04003649; recruiting;<br>PI: Behnam Badie		City of Hope Medical Center, USA	
IL13Rα2	Refractory malignant glioma	*IL13(E13Y)-CD3ζ<br>*Hy/TK selection/suicide	DNA electropora-tion, followed by drug selection	icr	*Up to 12 local infusions<br>*max. dose 10^8<br>*1.6x10^8 for first cycle +3x10^8 for second to fourth cycle (split dose in each cycle; 3 infusions 2 days apart)	phase 1	*actual enrollment: 3<br>*no preconditioning	*Brown et al. 2015: 3 patients, IL13(E13Y-zetakine)(scFv-CD3ζ) CD8+ T cells. Transient glioma responses observed in 2 of 3. Reduced IL13Rα2 expression after treatment (1P). Increase in tumor necrotic volume (1P).	NCT00730613; completed;<br>PI: Stephen Forman	[151,152]	City of Hope Medical Center, USA	*Well tolerated with manageble temporary brain inflammation.<br>*Grade 3 headache in two patients receiving 10^8 CAR-T cells.<br>*Grade 3 neurologic adverse events observed in 1 patient.<br>*On-target/off-tumor toxicity: neurologic event
IL13Rα2	Stage III or stage IV malignant glioma	GRm13Z40-2, an glucocorticoid-receptor-depleted allogeneic CD8+ cytolytic T-cell line expressing:<br>*IL13(E13Y)-CD3ζ<br>*Hy/TK selection/suicide<br>*[^{18}F]FHBG labeled	genetically modified?	it	?	phase 1	*actual enrollment: 6<br>*with IL-2	[18F]FHBG gene reporter allowed longitudinal imaging of intratumoral CAR-T	NCT01082926; completed;<br>PI: Behnam Badie	[153]	City of Hope Medical Center, USA	
IL13Rα2	Stage IIIC or IV melanoma	*IL13(E13Y)-hinge-optimized-4-1BB/CD3ζ<br>*truncated CD19	lentiviral	iv	Dose escalation	phase 1	*est. enrollment 24<br>*nonmyeloablative conditioning (cy+flu)<br>*w/ or w/o IL-2 subcutaneously		NCT04119024; Suspended (SAE review) ;<br>PI: Anusha Kalbasi		UCLA / Jonsson Comprehensive Cancer Center, USA	
IL13Rα2	Refractory malignant glioma	*IL13(E13Y)-hinge-optimized-4-1BB/CD3ζ<br>*truncated CD19<br>*memory enriched cells (Tcm)!	lentiviral (self-inactivating)	*stratum 1: it<br>*stratum 2: ic<br>*stratum 3: intraventricular<br>*stratum 4: dual delivery (it + in-traventricular)<br>*stratum 5: dual delivery (naive and memory T cells, via it + in-traventricular)	*determine MTD and a recommended phase II dosing plan (RP2D)	phase 1	*est. enrollment: 92<br>*no pretreatment	Brown et al. 2016:<br>Report on one patient: local control after intracavitary administration of 6 cycles of IL13-4-1BB/CD3ζ-CAR T. Distant disease foci progressed.<br>After 5 additional cycles (intraventricular): all tumors and metastases decreased by 77-100%. Response lasted 7.5 months, then four new lesions. (probably due to IL13Rα2 downregulation).	NCT02208362; recruiting;<br>PI: Behnam Badie	[154]	City of Hope Medical Center, USA	No CAR-T cell infusion related toxic effects of grade 3 or higher were observed<br>*Headaches, generalized fatigue, myalgia, olfactory auras<br>*On-target/off-tumor toxicity: no
Lewis Y	Solid tumors	scFv-CD28/CD3ζ	transduction ?	iv	MTD; single infusion 4 dose levels, each with 3 patients<br>*5 patients will get Indium^{111}-labeled T cells for tracking<br>*dose levels:-1 (if needed): 1x10^8<br>1: 2x10^8<br>2: 5x10^8<br>3: 1x10^9<br>4: 5x10^9	phase 1	*est. enrollment: 30<br>*cy+flu		NCT03851146; recruiting;<br>PI: Ben Solomon		Peter MacCallum Cancer Centre, Melbourne, Australia	
LMP1 (EBV)	Nasopharyngeal carcinoma	?	?	?	?	phase 1/2	*est. enrollment: 20		NCT02980315; unknown;<br>PI: not provided		Second Hospital of Nanjing Medical University, China	
mesothelin	*Non-small-cell Lung Cancer<br>*Mesothelioma	*αPD1-MSLN-CAR T cells<br>*secreting PD1 nanobodies	?	iv	*standard 3+3 dose escalation, single dose<br>*1×10^5 CAR+ T cells/kg,<br>*3×10^5 CAR+ T cells/kg,<br>*1×10^6 CAR+ T cells/kg,<br>*3×10^6 CAR+ T cells/kg	early phase 1	*est. enrollment: 10<br>*cyclophosphamide		NCT04489862; recruiting;<br>PI: Xiaorong Dong		*Shanghai Cell Therapy Group Co.,Ltd<br>*Wuhan Union Hospital, China	
mesothelin	r/r epithelial ovarian cancer	?	retroviral	?	?	phase 1/2	*est. enrollment: 20<br>*cy+flu		NCT03916679; recruiting;<br>PI: Jianwei Zhou		Second Affiliated Hospital, School of Medicine, Zhejiang University, China	
mesothelin	r/r ovarian cancer	4th generation	transduction ?	?	5x10^6/kg once at day 1	early phase 1	*est. enrollment: 10<br>*cy+flu		NCT03814447; recruiting;<br>PI: Hui Zhao		Shanghai 6th People's Hospital, China	
mesothelin	r/r ovarian cancer	?	retroviral	?	?	early phase 1	*est. enrollment: 20<br>*cy+flu		NCT03799913; recruiting;<br>PI: Jianwei Zhou		Zhejiang University, China	
mesothelin	Mesothelin+ multiple solid tumors	PD-1 gene knocked out (CRISPR/Cas9)!	?	?	dose escalation 3+3	phase 1	*est. enrollment: 10<br>*paclitaxel and cyclophosphamide		NCT03747965; recruiting;<br>PI: Weidong Han		Chinese PLA General Hospital, China	
mesothelin	Mesothelin expressing cancers:<br>*lung adenocarcinoma,<br>*ovarian,<br>*peritoneal,<br>*fallopian tube,<br>*mesothelioma pleural,<br>*mesothelioma peritoneum	*scFv-4-1BB/CD3ζ<br>*scFv based on SS1	lentiviral	iv, ip	*cohort 1 single dose 1-3x10^7/m^2 w/o cyclo<br>*cohort 2: single dose 1-3x10^7/m^2 w/ cyclo<br>*cohort 3: single dose 1-3x10^8/m^2 w/o cyclo<br>*cohort 4: single dose 1-3x10^8/m^2 w/ cyclo<br>*cohort 5: single dose 1-3x10^7/m^2 w/o cyclo IP<br>*3+3 dose escalation design to determine MTD	phase 1	*est. enrollment: 30<br>*with and w/o cyclophosphamide	In Tanyi et al.,:<br>Ovarian cancer single dose 3x10^7/m^2 w/o cyclo, 1/1 CR	NCT03054298; Active, not recruiting;<br>PI: Janos L Tanyi	[241]	Upenn, USA	Fever, high volume pleural fluid
mesothelin	HER2-negative, mesothelin-positive breast cancer	*iCasp9<br>*scFv based on m912	?	iv	MTD; dose escalation 3+3, single dose per patient	phase 1	*est. enrollment: 24<br>*cyclophosphamide		NCT02792114; recruiting;<br>PI: Prasad Adusumilli		MSKCC, USA	

Antigen	Cancer	CAR type /add-ons	CAR transfer	Mode of injection	Number of cells	Phase	Patient # /pretreatment /other drugs	Outcome	NCT# /Status /PI	Ref	Origin	Adverse events
mesothelin	Mesothelin+ advanced solid tumor	PD-1 antibody expressing	?	iv	two cycles	phase 1/2	*est. enrollment: 50<br>*cy+flu		NCT03615313; recruiting,;<br>PI: Juemin Fang, Song Gao, Yianling Guo, Hui Wang, Zhongzheng Zhu, Jianhua Chen		Shanghai 10th People's Hospital, China	
mesothelin	Mesothelin+ multiple solid tumors	Allogeneic cells, CRISPR-Cas9-mediated PD-1 and TCR gene-knocked out	?	?	Dose escalation, 3+3 dose escalation approach	phase 1	*est. enrollment: 10<br>*conditioning regimen		NCT03545815; recruiting;<br>PI: Han Weidong		Chinese PLA General Hospital, China	
mesothelin	Mesothelin+ advanced malignancies (adults)	PD1 antibody expressing	?	iv	dose escalation	phase 1/2	*est. enrollment: 40		NCT03030001; unknown;<br>PI: Qijun Qian		Shanghai Cell Therapy Engineering Research Institute, China	
mesothelin	Recurrent or metastatic malignant tumors	?	?	?	dose escalation $5x10^4$/kg-$1x10^7$/kg	phase 1	*est. enrollment: 20<br>*cyclophosphamide		NCT02930993; unknown;<br>PI: Jinwen Sun		China Meitan General hospital, China	
mesothelin	Mesothelin+ advanced solid tumors	CTLA-4 and PD-1 antibodies expressing	?	iv	2-$5x10^7$ cells/kg	phase 1/2	*est. enrollment: 40<br>*cyclophosphamide		NCT03182803; unknown;<br>PI: Jiangtao Wang		Ningbo No.5 Hospital, China	
mesothelin	Relapsed and/or chemotherapy refractory advanced malinancies:<br>*malignant mesothelioma,<br>*pancreatic cancer,<br>*ovarian,<br>*triple negative breast,<br>*endometrial,<br>*other mesothelin positive tumors	scFv-4-1BB/CD3ζ	retroviral	?	dose escalation 3+3	phase 1	*est. enrollment: 20		NCT02580747; unknown;<br>PI: Han Weidong		Chinese PLA General Hospital, China	
mesothelin	MSTL+ metastatic cancer:<br>*cervical,<br>*pancreatic,<br>*ovarian,<br>*lung cancer,<br>*mesothelioma	?	retroviral	iv	Phase 1: dose escalation until MTD determined, then phase 2<br>Phase 2 not reached<br>D1: $1x10^6$<br>D2: $3x10^6$<br>D3: $1x10^7$<br>D4: $3x10^7$<br>D5: $1x10^8$	phase 1/2	*est. enrollment: 136<br>*actual recruitment: 15<br>*non-myeloablative, but lymphoid depleting regimen (cy+flu)<br>*co-treatment: low-dose aldesleukin (IL-2, also iv (72,000IU/kg));	*1 SD (DL: $3x10^6$)<br>*all other 14 PD<br>*not that many SAE	NCT01583686; Terminated (Study terminated due to slow/insufficient accrual.);<br>PI: SA Rosenberg		NCI, NIH, USA	
mesothelin	Mesothelin expressing cancers:<br>*metastatic pancreatic,<br>*serous epithelial ovarian,<br>*pleural mesothelioma	*scFv-4-1BB/CD3ζ<br>*scFv based on SS1	lentiviral	iv	1-$3x10^7/m^2$ (cohort 1 and 2)<br>1-$3x10^8/m^2$ (cohort 3 and 4)	phase 1	*19<br>*with and without cyclophosphamide	*treatment well tolerated<br>*6 of 6 SD	NCT02159716; completed;<br>PI: Haas	[242]	Upenn, USA	
mesothelin	Pancreatic cancer	*scFv-4-1BB/CD3ζ<br>*scFv based on SS1<br>*combined with anti-CD19 scFv-4-1BB/CD3ζ CAR	lentiviral	iv	1-$3x10^7/m^2$ (cohort 1) or 1-$3x10^8/m^2$ (cohort 2) CAR-T-positive cells	phase 1	*actual enrollment: 4<br>*cyclophosphamide		NCT02465983; Terminated (Lack of efficacy and funding to continue investigation);<br>PI: Ko		*Upenn<br>*University of California, San Francisco, USA	
mesothelin	Metastatic pancreatic cancer	scFv-4-1BB/CD3ζ	lentiviral	?	dose escalation (DLT)<br>cohort 1: single dose of 1-$3x10^7/m^2$ CAR+ cells<br>cohort 2: single dose of 1-$3x10^8/m^2$ CAR+ cells		*est. enrollment: 10<br>*one dose of cyclophosphamide		NCT03638193; recruiting;<br>PI: Jinfei Chen		The first affiliated hospital with Nanjing medical university, China	
mesothelin	Malignant pleural disease:<br>*mesothelioma,<br>*metastatic lung,<br>*breast cancers	*scFv-CD28/CD3ζ<br>*iCasp9	genetically engineered?	ip	*dose escalation<br>*maximum dose of $6x10^7$ cells/kg, or until MTD is reached	phase 1	*est. enrollment: 48<br>*with and without cyclophosphamide	2/14 CR, 5/14 PR, 4/14 SD	NCT02414269; recruiting;<br>PI: Adusumilli	[243]	MSKCC, USA	
mesothelin	Pancreatic cancer	humanized scFv	lentiviral	iv	DLT, 3 cohorts, 3+3 dose escalation design<br>*cohort 1: single dose 1-$3x10^7/m^2$ cells, without cyclophosphamide<br>*cohort 2: single dose 1-$3x10^8/m^2$ cells, without cyclophosphamide<br>*cohort 3: single dose 1-$3x10^8/m^2$ cells, with cyclophosphamide	phase 1	*est. enrollment: 18<br>*with and without cyclophosphamide		NCT03323944; Active, not recruiting ;<br>PI: Mark O'Hara		Upenn, USA	
mesothelin	Advanced pancreatic carcinoma	scFv-4-1BB/CD3ζ	transduction ?	vascular intervention, TAI	1-$10x10^6$ mesoCAR positive T cells/kg	phase 1	*est. enrollment: 30<br>*cyclophosphamide		NCT02706782; unknown;<br>PI: Xu Aimin		RenJi Hospital, China	
mesothelin	Pancreatic cancer	*murine-derived scFv-4-1BB/CD3ζ<br>*CD19 -CAR	lentiviral	pa or iv	?	early phase 1	*est. enrollment: 10<br>*cyclophosphamide		NCT03497819; active, not recruiting;<br>PI: Mengtao Zhou		First affiliated hospital of Wenzhou medical university, China	

Antigen	Cancer	CAR type /add-ons	CAR transfer	Mode of injection	Number of cells	Phase	Patient # /pretreatment /other drugs	Outcome	NCT# /Status /PI	Ref	Origin	Adverse events
Mesothelin	*Malignant pleural mesotheliomas (MPM)<br>*Metastatic pancreatic cancer (PDA)	*scFv-4-1BB/CD3ζ<br>*scFv based on ss1 (murine Ab)	mRNA-transfection	iv, it	*In Beatty et al., 2014:<br>*MPM: 1x10^8 iv, 1x10^9 iv, 1x10^8 iv<br>*PDA: 8 times 3x10^8/m^2 iv over 18 days, then 2 times 2x10^8 i.t.<br>*In Maus et al.,:<br>cohort 1: 1x10^8 iv day 0 then 1x10^9 iv day 7<br>cohort 2: 1x10^8 iv day 0,2,4 then 1x10^9 iv day 7,9,11	phase 1	In Beatty et al., 2014:<br>*2 case reports of ongoing trial<br>*no lymphodepletion<br>In Maus et al.,:<br>*18<br>*pretreatment: none	In Beatty et al., 2014:<br>*Cells migrated to primary and metastatic tumor sites<br>*Clinical and laboratory evidence of antitumor activity in both patients<br>*Cell elicited antitumor immune response revealed by the development of novel anti-self antibodies<br>*Induction of (humoral) epitope spreading<br>*no evidence of off-tumor on-target toxicity against normal tissues<br>*2/2SD<br>*Note: MPM patient hat adverse events: cardiac arrest respiratory failure disseminated intravenous coagulation, cytokine release syndrom at study day 42 (= within 24h of last infusion!) is the same patient as described in Maus et al.<br>In Maus et al.,:<br>*In one out of 4 patients anaphylaxis and cardiac arrest within minutes of completing the third infusion<br>*CAR based on murine antibody; probably AE caused by IgE antibodies specific to the CAR<br>*CARs based on murine antibodies may be a safety issue for mRNA CARs, especially when administered using an intermittent dosing schedule<br>*1/4 CR, 1/4 PD, 2/4 SD, 1/4 died	NCT01355965; completed;<br>PI: Haas	[223,225]	Upenn, USA	*One patient reported minimal arthralgia and fatigue<br>*Anaphylactic reaction in one patient, leading to grade 4 cardiac arrest, respiratory failure, disseminated intravenous coagulation, and CRS<br>*Grade 4 jejunal obstruction, grade 3 abdominal pain, and grade 2 lymphcytosis for another patient<br>*On-target/off-tumor toxicity: no
Mesothelin	*Metastatic pancreatic cancer (PDA) (pancreatic ductal adenocarcinoma (PDAC))	*scFv-4-1BB/CD3ζ<br>*scFv based on ss1 (murine Ab)	mRNA-transfection	iv, it	1 to 3x10^8/m^2, three times weekly, for three weeks	phase 1	*16<br>*pretreatment: none	*no cytokine release syndrome<br>*no dose-limiting toxicities<br>*SD in 2 patients<br>*total metabolic active volume (MAV) remained stable in 3 patients and decreased by 69.2% in 1 patient	NCT01897415; completed;<br>PI: Beatty	[224]	Upenn, USA	*Grade > 3 toxicities included abdominal pain (1) and backpain (1)<br>*On-target/off-tumor toxicity: no
mesothelin	*Advanced ovarian cancer<br>*Peritoneal mesothelioma	MCY-M11	mRNA-transfection	ip	dose escalation<br>3 weekly doses	phase 1	est. enrollment: 15		NCT03608618; recruiting;<br>PI: Claudio Dansky Ullmann		Maxcyte Inc., USA	
MG7 (glycosylased form of CEA)	MG7+ liver metastases	scFv-4-1BB/CD3ζ	transduction ?	it	1-6x10^8 CAR+ T cells	phase 1/2	*est. enrollment: 20<br>*cyclophosphamide		NCT02862704; unknown;<br>PI: Yongzhan Nie		Xijing Hospital of digestive diseases, China	
MUC1	Esophageal cancer	PD-1 knockout	?	?	?	phase 1/2	est. enrollment: 20		NCT03706326; recruiting;<br>PI: not provided		The first affiliated hospital of Guangdong Pharmaceutical University, China	
MUC1	Intrahepatic cholangiocarcinoma	?	?	?	?	phase 1/2	*est. enrollment: 9<br>*cy+flu		NCT03633773; recruiting;<br>PI: not provided		Second Affiliated Hospital, School of Medicine, Zhejiang University, China	
MUC1	MUC1+ advanced solid tumors	CTLA-4 and PD-1 antibodies expressing	genetically engineered?	iv	2-5x10^7 cells/kg	phase 1/2	*est. enrollment: 40<br>*cyclophosphamide		NCT03179007; unknown;<br>PI: Jiangtao Wang		Ningbo No.5 Hospital, China	
MUC1	NSCLC	PD1 knockout	genetically engineered?	infused?	?	phase 1/2	est. enrollment: 60		NCT03525782; recruiting;<br>PI: Size Chen		First affiliated hospital of Guangdong Pharmaceutical University, China	
MUC1	MUC1+ solid tumor:<br>*malignant glioma,<br>*colorectal,<br>*gastric	?	transduction ?	infusion?	?	phase 1/2	est. enrollment: 20		NCT02617134; unknown;<br>PI: Lin Yang		*PersonGen BioTherapeutics (Suzhou)<br>*The first people's hospital of Hefei, China	
Muc1	MUC1+ advanced refractory solid tumor:<br>*hepatocellular carcinoma,<br>*NSCLC,<br>*pancreatic carcinoma,<br>*triple-negative invasive breast carcinoma	*scFv-CD28/4-1BB/CD3ζ<br>*scFv based on SM3 or pSM3	lentiviral	it	5x10^5 cells per lesion	phase 1/2	est. enrollment: 20<br>*no preconditioning	*1/1 PR<br>*pSM3-CAR treated tumor lesion showed necrosis	NCT02587689; unknown;<br>PI: Lin Yang	[244]	*PersonGen BioTherapeutics (Suzhou)<br>*The first people's hospital of Hefei, China	*Mild headache, muscle pain, nasal congestion, and abdominal bloating discomfort, and a transient CRSwas experienced<br>*On-target/off-tumor toxicity: no
MUC16ecto	Recurrent MUC16ecto+ solid tumors:<br>*ovarian,<br>*peritoneal or<br>*fallopian tube	*scFv-CD28/CD3ζ<br>*express also IL-12<br>*EGFRt<br>*scFv based on 4H11	?	iv and ip	3x10^5, 1x10^6, 3x10^6, 1x10^7 CAR+ T cells/kg	phase 1	*est. enrollment: 30<br>*cyclophosphamide with/without fludarabine		NCT02498912; recruiting;<br>PI: Roisin O'Cearbhaill	[139]	MSKCC, USA	
Muc1* (cleaved form of Muc1)	Breast cancer	*scFv-CD8 hinge-4-1BB/CD3ζ<br>*huMNC2-CAR44<br>*humanized MNC2	lentiviral	?	*MTD and RP2D<br>*3+3	phase 1	*est. enrollment: 69		NCT04020575; recruiting;<br>PI: Jennifer M Specht		Fred Hutchinson Cancer Research Center, USA	

Antigen	Cancer	CAR type<br>/add-ons	CAR transfer	Mode of injection	Number of cells	Phase	Patient #<br>/pretreatment<br>/other drugs	Outcome	NCT#<br>/Status<br>/PI	Ref	Origin	Adverse events
TnMuc1	Advanced solid tumors:<br>*NSCLC,<br>*ovarian,<br>*fallopian tube,<br>*TNBC,<br>*pancreatic ductal adenocarcinoma and<br>*multiple myeloma	?	?	iv	dose escalation	phase 1	*est. enrollment: 80<br>*lymphodepletion cy+flu		NCT04025216;<br>Active, not recruiting;<br>PI: not provided		Tmunity Therapeutics, USA	
Nectin4/FAP	Malignant solid tumors:<br>*NSCLC,<br>*breast,<br>*ovarian,<br>*bladder,<br>*pancreatic	*4th generation<br>*IL-7 and CCL19 or/and IL-12 expressing	?	it	?	phase 1	*est. enrollment: 30		NCT03932565;<br>recruiting;<br>PI: Bingmu Fang, M.D	[180-182]	*The Sixth Affiliated Hospital of Wenzhou Medical University<br>*Lishui Country People's Hospital, China	
NKG2D-Ligands (MIC-A, MIC-B, and the ULBPs 1 to 6	*Colorectal cancer<br>*TNBC<br>*Sarcoma<br>*Nasopharyngeal carcinoma<br>*Prostate cancer<br>*Gastric cancer	*Haplo/allogeneic gamma/delta T cells<br>*CTM-N2D-101	?	iv	*dose escalation<br>*four times weekly<br>*DL1: $3x10^8$<br>*DL2: $1x10^9$<br>*DL3: $3x10^9$	phase 1	*est. enrollment: 10		NCT04107142;<br>not yet recruiting;<br>PI: not provided		CytoMed Therapeutics Pte Ltd, Malaysia	
NKG2D-Ligands (MIC-A, MIC-B, and the ULBPs 1 to 6)	Colorectal cancer with potentially resectable liver metastases	NKG2D-CD3ζ (NKR-2)	?	iv	*Dose escalation and dose expansion<br>*3 dose levels, per dose 3 successive administrations two weeks apart	phase 1	*est. enrollment: 36<br>*concurrently with FOLFOX	multiple iv w/ concurrent FOLFOX:<br>1/3 PR	NCT03310008;<br>active, not recruiting;<br>PI: Frederic Lehmann	[42]	Celyad, Belgium	
NKG2D-Ligands (MIC-A, MIC-B, and the ULBPs 1 to 6)	Multiple cancer indications:<br>*colorectal,<br>*ovarian,<br>*bladder,<br>*triple-negative breast,<br>*pancreatic,<br>*myeloid leukemia and<br>*multiple myeloma	NKG2D-CD3ζ (NKR-2)	?	iv	*Dose escalation<br>*3 dose levels, per dose 3 successive administrations two weeks apart	phase 1/2	*est. enrollment: 122<br>*w/ or w/o preconditioning	multiple iv w/o preconditioning:<br>4/14 SD (3 colon, 1 ovarian)	NCT03018405;<br>recruiting;<br>PI: Frederic Lehmann	[42,43]	Celyad, Belgium/USA	
NKG2D-Ligands (MIC-A, MIC-B, and the ULBPs 1 to 6)	Non-resectable liver metastases from colorectal cancer	NKG2D-CD3ζ (NKR-2)	?	iha	Multiple administrations;<br>dose escalation:<br>3 dose levels, per dose 3 successive HTAs two weeks apart	phase 1	*est. enrollment: 18		NCT03370198;<br>active, not recruiting;<br>PI: Frederic Lehmann		Celyad, Belgium	
NKG2D-Ligands (MIC-A, MIC-B, and the ULBPs 1 to 6)	Colorectal cancer	*Allogeneic NKG2D-based CYAD-101<br>*standard chemotherapy regimen and immunotherapy with allogenic CAR-T	?	iv	*3 doses, dose escalation (3+3 design)<br>cohort 1: $1x10^8$ per injection<br>cohort 2: $3x10^8$ per injection<br>cohort 3: $1x10^9$ per injection	phase 1	*est. enrollment: 36<br>*concurrently with FOLFOX		NCT03692429;<br>recruiting;<br>PI: Fiona Thistlethwaite, Sophie Paoa, Richard Kim		Celyad, USA/UK	
NKG2D-Ligands (MIC-A, MIC-B, and the ULBPs 1 to 6)	*HCC<br>*Glioblastoma<br>*Medulloblastoma<br>*Colon cancer	*KD-025 CAR T-cells<br>*NKG2D-CD8 hinge/TM-4-1BB/CD3ζ	transduction ?	iv or HPA injection	Dosage:1-$10x10^6$/kg, 70ml/time, over 20-30 minutes; single injection	Phase 1/2	est. enrollment: 10		NCT04270461;<br>not yet recruiting;<br>PI: Changchun Cai		Affiliated hospital of jiujiang university, Jiujiang, Jiangxi, China	
Ovarian cancer	Ovarian cancer	IgT cells (modified with immune modulatory genes such as genes encoding immune checkpoint inhibitors; PD1 and PD-L1 antibodies)	?	iv or it	?	phase 1/2	*est. enrollment: 100		NCT03184753;<br>recruiting;<br>PI: Lung-Ji Chang		Shenzhen Geno-Immune Medical Institute, China	
PD-L1	NSCLC	scFv-4-1BB/CD3ζ	lentiviral	?	*total number is 1-$2x10^6$/kg	phase 1	*est. enrollment: 22<br>*cy+flu		NCT03330834;<br>recruiting;<br>PI: Li Zhang		Sun Yat-sen University, China	
PD-L1	Recurrent glioblastoma multiforme	*Chimeric Switch receptor (CSR)<br>*PD1-CD28 (switches T cells on after binding to PD-L1 on tumor)<br>*tEGFR	?	iv	*3+3 dose escalation<br>*$5x10^4$/kg-$1x10^7$/kg	phase 1	*est. enrollment: 20<br>*cy+flu		NCT02937844;<br>unknown;<br>PI: not provided		Beijing Sanbo Brain Hospital, China	
PSCA	Metastatic castration resistant prostate cancer	*scFv-4-1BB/CD3ζ<br>*tCD19	virally transduced	iv	dose-escalation	phase 1	*est. enrollment: 33<br>*cy+flu		NCT03873805;<br>recruiting;<br>PI: Tanya B Dorff		City of Hope Medical Center, USA	
PSCA	Selected advanced solid tumors:<br>*pancreatic,<br>*stomach,<br>*prostate cancers	*scFv-CD28/CD3ζ<br>*CAR-T have iMC	retroviral	?	MTD<br>escalating dosis: from $1.25x10^6$ cells/kg up to $5.0x10^6$ cells/kg	phase 1/2	*est. enrollment: 30<br>*cy+flu	8/15 SD, 3/15 PD	NCT02744287;<br>recruiting;<br>PI: Carlos Becerra	[207]	Baylor health care system, USA	
PSMA	Refractory castrate-resistant prostate cancer	?	lentiviral	iv	*3+3 dose escalation<br>*cohort 1: single dose 0.5-$1x10^7$/kg<br>*cohort 2: single dose 3-$6x10^7$/kg	phase 1	*est. enrollment: 12<br>*cy+flu		NCT04053062;<br>recruiting;<br>PI: Shancheng Ren		Changhai Hospital, China	
PSMA	Metastatic castration-resistant prostate cancer	Dominant negative TGFβ receptor	genetically modified?	iv	?	phase 1	*est. enrollment: 18<br>*cy+flu		NCT04227275;<br>recruiting;<br>PI: ?		*Columbia University Medical Center, New York<br>*Thomas Jefferson University, Philadelphia, USA	
PSMA	Metastatic castration-resistant prostate cancer	*P-PSMA-101 CAR<br>*CARTyrin (nanobody (Centyrin))-CD28/CD3ζ<br>*In Tscm (stem cell memory) cells<br>*iCasp9	PiggyBac transposon	iv	*3 + 3 design of dose-escalating cohorts of single and multiple doses	phase 1/2	*est. enrollment: 40<br>*Drug: Rimiducid (safety switch activator)<br>*lymphodepleting chemotherapy		NCT04249947;<br>recruiting;<br>PI: Matthew Spear		*City of Hope Comprehensive Cancer Center, Duarte, USA<br>*Sarah Cannon Research Institute at HealthONE Denver<br>*Memorial Sloan Kettering Cancer Center New York	

Antigen	Cancer	CAR type /add-ons	CAR transfer	Mode of injection	Number of cells	Phase	Patient # /pretreatment /other drugs	Outcome	NCT# /Status /PI	Ref	Origin	Adverse events
PSMA	Castrate-resistant metastatic prostate cancer	*scFv-CD28/CD3ζ<br>*scFv based on J591	retroviral	?	DLT<br>DL1: 1x10^{7} CAR+ T cells/kg<br>DL2: 3x10^{7} CAR+ T cells/kg<br>DL3: 1x10^{8} CAR+ T cells/kg	phase 1	*actual enrollment: 13<br>*single dose of cyclophosphamide		NCT01140373; active, not recruiting;<br>PI: Susan Slovin		MSKCC, USA	
PSMA	Castrate-resistant prostate cancer	*2nd generation<br>*Dominant negative form of TGFbeta receptor	lentiviral	iv	*3+3 dose escalation<br>*cohort 1: 1-3x10^{7}/m^{2} without cyclo<br>*cohort 2: 1-3x10^{8}/m^{2} without cyclo<br>*cohort 3: MTD with cyclo	phase 1	*est. enrollment: 18<br>*with and without cyclophosphamide		NCT03089203; recruiting;<br>PI: Naomi Haas	[195]	Upenn, USA	
PSMA	Advanced prostate cancer	?	retroviral	iv	single dose 10^{10}-10^{11}	phase 1	*est. enrollment: 18<br>*non-myeloablative conditioning<br>*low or moderate IL-2		NCT00664196; suspended (funding);<br>PI: Richard P Junghans		Roger Williams Medical Center, USA	
PSMA	Prostate cancer	*scFv-CD8hinge-CD3ζ<br>*scFv based on 3D8	retroviral	iv	single dose 10^{9} or 10^{10}	phase 1	*est. enrollment: 12<br>*non-myeloablative conditioning cy+flu<br>*low or moderate IL-2	2/5 PR but relapsed after 1–2 months, 1/5 minor response, 2/5 NR	NCT01929239; suspended (funding);<br>PI: Richard P Junghans, PhD, MD	[245,246]	Roger Williams Medical Center, USA	*Grade 3/4 hematologic toxicities including neutropenia, neutropenic fever, and thrombocytopenia in all the patients; anemia, hypocalcemia, hypophosphatemia, and appendicitis in one patient.<br>*Grade 1/2 skin rash, fatigue, intermittent low-grade fevers, and muscle pain in some patients.<br>*On-target/off-tumor toxicity: no
PSMA	PSMA+ tumors:<br>*prostate,<br>*brain tumor,<br>*neuroblastoma	4SCAR	?	iv	10^{6} cells/kg	phase 1/2	*est. enrollment: 100		NCT04429451; recruiting;<br>PI: ?		*Shenzhen Children's Hospital<br>*Shenzhen Geno-immune Medical Institute<br>*Shenzhen Hospital of Southern Medical University<br>*The Seventh Affilliated Hospital of Sun Yat-Sen<br>*University Shenzhen, Guangdong, China	
ROR1	Advanced ROR1+ malignancies:<br>*CLL, MCL, ALL,<br>*NSCLC,<br>*TNBC	scFv-4-1BB/CD3ζ	genetically modified?	iv	dose escalation<br>3.3x10^{5}-1x10^{7} cells/kg	phase 1	*est. enrollment: 60<br>*cy+flu	AACR 2018: 4/5 MR (2 NSCLC, 2 TNBC), 1/5 SD (TNBC)<br><br>San Antonio Breast Cancer Symposium (SABCS) 2018: 2/4 SD, 1/4 PR	NCT02706392; Suspended (COVID19 Suspension);<br>PI: David Maloney	[247,248]	Fred Hutchinson Cancer Research Center, USA	
ROR2	*Solid tumor<br>*Soft tissue sarcoma<br>*Gastric cancer<br>*Pancreatic cancer<br>*Bladder cancer	CCT301-59 CAR T	?	iv	dose escalation<br>3+3<br>one dose<br>DL1: 1x10^{6} CAR+ T cells/kg<br>DL2: 3x10^{6} CAR+ T cells/kg<br>DL3: 1x10^{7} CAR+ T cells/kg	phase 1	*est. enrollment: 18<br>*cy+flu		NCT03960060; recruiting;<br>PI: Yuhong Zhou		Shanghai Zhongshan Hospital, China	
VEGFR2	*Metastatic melanoma<br>*Renal cancer	?	retroviral	iv	MTD determination<br>Dose escalation:<br>1x10^{6}-3x10^{10} cells	phase 1/2	*actual enrollment: 24<br>*lymphodepletion: cy+flu<br>*CAR-T with aldesleukin (IL-2)	0 CR1 PR (DL: 1x10^{10} cells (low dose IL-2))<br>22 PD<br>1 SD (DL: 1x10^{9} cells (high dose IL-2))	NCT01218867; Terminated (No objective responses were observed?);<br>PI: Steven Rosenberg		NCI, NIH, USA	*Grade 3/4 toxicity include nausea, vomiting, hypoxia, and elevated levels of aspartate transaminase, alanine transaminase, and bilirubin<br>*23 recipients experienced blood and lymphatic system disorders.<br>*More than 4 participants suffered from gastrointestinal events.<br>*Two suffered from fever<br>.*8 afflicted by fatigue.<br>*Seven suffered from dyspnea.<br>*Eight suffered from hypoalbuminemia.
c-MET/PD-L1	HCC	?	lentiviral	iv	3 injections<br>2x10^{6}/kg	phase 1	*est. enrollment: 50<br>*cy+flu or Bis-1-nitrosourea+Etoposide+Arabinoside+cyclophosphamide		NCT03672305; not yet recruiting;<br>PI: Guozhong Ji		Second affiliated hospital of nanjing medical university, China	
mesothelin, PSCA, CEA, HER2, MUC1, EGFRvIII, and other?	Pancreatic cancer	?	?	iv	single dose1x10^{7}/kg	early phase 1	est. enrollment: 10		NCT03267173; unknown;<br>PI: Wie Yunwei		First affiliated hospital of Harbin medical university, China	
GD2, PSMA, MUC1, mesothelin or other	Cervical cancer	?	?	iv	1 infusion:<br>1x10^{6}-1x10^{7} cells/kg	phase 1/2	*est. enrollment: 20		NCT03356795, recruiting,<br>PI: Lung-Ji Chang		Shenzhen Geno-Immune Medical Institute, China	
CD133, GD2, MUC1, CD117 or other	Sarcoma	4SCART	?	iv	1 infusion:<br>1x10^{6}-1x10^{7} cells/kg	phase 1/2	*est. enrollment: 20		NCT03356782; recruiting;<br>PI: Lung-Ji Chang	[41]	Shenzhen Geno-Immune Medical Institute, China	
PSMA and folate receptor alpha (FRA)	Bladder cancer	4SCART	?	?	?	phase 1/2	*est. enrollment: 20		NCT03185468; recruiting;<br>PI: Lung-Ji Chang		Shenzhen Geno-Immune Medical Institute, China	
PD-L1 and CD80/86	Refractory/relapsed NSCLC	Zeushield Cytotoxic T Lymphocytes (Z-CTLs)	?	?	Dose level one:<br>1.0x10^{5} cells/kg<br>Dose level two:<br>1.0x10^{6} cells/kg<br>Dose level three:<br>1.0x10^{7} cells/kg	early phase 1	est. enrollment: 10		NCT03060343; unknown;<br>PI: Yu Fenglei and Li Peng		Second Xiangya Hospital of Central South University, China	
GPC3, mesothelin, CEA	hepatocellular carcinoma (GPC3)<br>pancreatic cancer (mesothelin)<br>colorectal cancer (CEA)	?	lentiviral	vascular interventional therapy or it	1.25-4x10^{7} CAR+ T cells/cm^{3} tumor bulk	phase 1/2	*est. enrollment: 20		NCT02959151; unknown;<br>PI: Wentao Li		Fudan University, China	
ROR2 or AXL	Stage IV renal cell carcinoma	*CCT301-59 when ROR2 positive<br>*CCT301-38 when ROR2 negative but AXL positive	?	iv	dose escalation and expansion<br>3 doses will be tested:<br>1x10^{5}/kg<br>1x10^{6}/kg<br>1x10^{7}/kg CAR+ T cells	phase 1/2	*est. enrollment: 66<br>*cy+flu		NCT03393936; active, not recruiting;<br>PI: not provided		Shanghai Sinobioway Sunterra Biotech, China	

Antigen	Cancer	CAR type<br>/add-ons	CAR transfer	Mode of injection	Number of cells	Phase	Patient #<br>/pretreatment<br>/other drugs	Outcome	NCT#<br>/Status<br>/PI	Ref	Origin	Adverse events
EGFRVIII, IL13Rα2, Her-2, EphA2, CD133, GD2	Recurrent malignant glioma	?	lentiviral	?	?	phase 1	est. enrollment: 100		NCT03423992;<br>recruiting;<br>PI: Qingtang Lin, Feng Ling		Xuanwu Hospital, Beijing, China	
CD19, BCMA, GPC3, CLD18.2	*B-cell leukemia/lymphoma,<br>*myeloma,<br>*HCC,<br>*pancreatic carcinoma and<br>*adenocarcinoma of esophagogastric junction	?	lentiviral	iv	3+3 dose escalation	?	*est. enrollment: 48<br>*cy+flu		NCT03302403;<br>recruiting;<br>PI: Mengtao Zhou, Kang Yu, Songfu Jiang		FirstAffiliated Hospital of Wenzhou Medical University, China	
HER2, mesothelin, Lewis-Y, PSCA, MUC1, PD-L1, CD80/86	Lung cancer	3rd generation	transduction ?	iv or it	?	phase 1	est. enrollment: 30		NCT03198052;<br>recruiting;<br>PI: Zhenfeng Zhang	[35]	Second Affiliated Hospital of Guangzhou Medical University, China	
MAGE-A1, MAGE-A4, MUC1, GD2, mesothelin, novel cancer antigens	Lung cancer	?	lentiviral	iv	1 infusion:<br>$1x10^6$-$1x10^7$ cells/kg	phase 1/2	*est. enrollment: 20		NCT03356808;<br>recruiting;<br>PI: Lung-Ji Chang		Shenzhen Geno-Immune Medical Institute, China	
NY-ESO-1, DR5, EGFRvIII, mesothelin	*Esophagus cancer<br>*Hepatoma<br>*Glioma<br>*Gastric cancer	?	?	?	?	phase 1/2	*est. enrollment: 50<br>*cy + flu		NCT03941626;<br>recruiting;<br>PI: not provided		Henan Provincial Hospital Shenzhen BinDEBio Ltd., China	
Transmembrane 4 L Six Family Member 1 (TM4SF1) or Epithelial cell adhesion molecule (EpCAM)	Refractory/recurrent advanced:<br>*pancreatic cancer,<br>*colorectal cancer,<br>*gastric cancer or<br>*lung cancer	?	?	iv	three escalating doses, 2.0-2.5, 4.0-5.0, and 8.0-10.0 ($\times10^6$/kg)	?	est. enrollment: 72		NCT04151186;<br>not yet recruiting;<br>PI: Helong Zhang	[36]	Suhaichuan, Tang-Du Hospital , China	
CD19, CD22, CD33, BCMA, CD38, NY-ESO-1, DR5, c-Met, EGFRvIII, mesothelin	Many different tumors incl.<br>melanoma (target NY-ESO-1)	?	?	?	?	phase 1/2	*est. enrollment: 73<br>*cy+flu		NCT03638206;<br>recruiting;<br>PI: ZhongHua Yang		Shenzhen BinDEBio Ltd., China	
Confirmed surface antigens including GD2, PSMA, Her2, CD276 or other markers	*Sarcoma<br>*Osteoid sarcoma<br>*Ewing sarcoma	CAR-T followed by sarcoma vaccines	?	iv	1 infusion, CAR-T $1x10^6$-$1x10^7$ cells/kg iv and vaccine 1-$5x10^6$ irradiated cells via subcutaneous injection	phase 1/2	*est. enrollment: 20<br>*with low dose chemotherapy		NCT04433221;<br>recruiting;<br>PI: ?		*The Seventh Affilliated Hospital, Sun Yat-Sen University<br>*Shenzhen Children's Hospital<br>*Shenzhen Geno-Immune Medical Institute, China	
Cancer specimens positive for any one or more of tumor-associated antigens, such as GD2, mesothelin, P16, MMP, Melan A, MAGE A1, MAGE A3, and MAGE A4	Cancer	EIE (engineered immune effector) cells including chimeric antigen receptor (CAR) modified immune cells	?	iv, it	?	phase 1/2	est. enrollment: 100		NCT03535246;<br>recruiting;<br>PI: ?		*QiFu Hospital of Guangzhou University of Chinese Medicine<br>*Shenzhen Geno-immune Medical Institute<br>*Yunnan Cancer Hospital & The Third Affiliated Hospital of Kunming Medical University & Yunnan Cancer Center, China	
GD2, Her2, and CD44v6	Breast cancer	4SCART (multiple)	?	iv	?	phase 1/2	*est. enrollment: 100		NCT04430595;<br>recruiting;<br>PI: ?		*The Seventh Affilliated Hospital, Sun Yat-Sen University<br>*Shenzhen Geno-Immune Medical Institute, China	
Undisclosed antigen	Advanced, recurrent platinum resistant<br>*ovarian,<br>*fallopian tube or<br>*primary peritoneal Cancer	*PRGN-3005 UltraCAR-T<br>*membrane bound IL-15<br>*kill switch for better control	sleeping beauty	ip and iv	*3+3 dose escalation	phase 1	*est. enrollment: 41		NCT03907527;<br>recruiting;<br>PI: John Liao	[189]	*Fred Hutch<br>*University of Washington Cancer Consortium, USA	
